# The in vitro and in vivo depigmentation activity of coenzyme Q_0_, a major quinone derivative from *Antrodia camphorata*, through autophagy induction in human melanocytes and keratinocytes

**DOI:** 10.1186/s12964-024-01537-6

**Published:** 2024-02-26

**Authors:** You-Cheng Hseu, Jou-Tsen Yeh, Chithravel Vadivalagan, Siang-Jyun Chen, Yugandhar Vudhya Gowrisankar, Sudhir Pandey, Yuan-Tai Hsu, Hung-Rong Yen, Hui-Chi Huang, Jhih-Hsuan Hseu, Hsin-Ling Yang

**Affiliations:** 1 Department of Cosmeceutics, College of Pharmacy, China Medical University, Taichung, 406040 Taiwan; 2 Chinese Medicine Research Center, China Medical University, Taichung, 406040 Taiwan; 3 Research Center of Chinese Herbal Medicine, China Medical University, Taichung, 406040 Taiwan; 4 Department of Health and Nutrition Biotechnology, Asia University, Taichung, 413305 Taiwan; 5 Institute of Nutrition, College of Health Care, China Medical University, Taichung, 406040 Taiwan; 6 Department of Surgery, University of Michigan Medical Center, Ann Arbor, Michigan 48109 United States; 7 Department of Medical Research, China Medical University Hospital, Taichung, 404333 Taiwan; 8 School of Chinese Medicine, China Medical University, Taichung, 404333 Taiwan; 9 Department of Chinese Pharmaceutical Sciences and Chinese Medicine Resources, College of Chinese Medicine, China Medical University, Taichung, 406040 Taiwan; 10 Department of Dermatology, Kaohsiung Chang Gung Memorial Hospital, Kaohsiung, 83301 Taiwan

**Keywords:** CoQ_0_, α-MSH, Autophagy, Melanogenesis, Melanin degradation

## Abstract

**Background:**

Coenzyme Q_0_ (CoQ_0_), a novel quinone derivative of *Antrodia camphorata*, has been utilized as a therapeutic agent (including antioxidant, anti-inflammatory, antiangiogenic, antiatherosclerotic, and anticancer agents); however, its depigmenting efficiency has yet to be studied.

**Methods:**

We resolved the depigmenting efficiency of CoQ_0_ through autophagy induction in melanoma (B16F10) and melanin-feeding keratinocyte (HaCaT) cells and in vivo Zebrafish model. Then, MPLC/HPLC analysis, MTT assay, Western blotting, immunofluorescence staining, LC3 transfection, melanin formation, GFP-LC3 puncta, AVO formation, tyrosinase activity, and TEM were used.

**Results:**

CoQ_0_-induced autophagy in B16F10 cells was shown by enhanced LC3-II accumulation, ATG7 expression, autophagosome GFP-LC3 puncta, and AVOs formation, and ATG4B downregulation, and Beclin-1/Bcl-2 dysregulation. In α‐MSH-stimulated B16F10 cells, CoQ_0_ induced antimelanogenesis by suppressing CREB-MITF pathway, tyrosinase expression/activity, and melanin formation via autophagy. TEM data disclosed that CoQ_0_ increased melanosome-engulfing autophagosomes and autolysosomes in α‐MSH-stimulated B16F10 cells. CoQ_0_-inhibited melanogenesis in α‐MSH-stimulated B16F10 cells was reversed by pretreatment with the autophagy inhibitor 3-MA or silencing of LC3. Additionally, CoQ_0_-induced autophagy in HaCaT cells was revealed by enhanced LC3-II accumulation, autophagosome GFP-LC3 puncta and AVO formation, ATG4B downregulation, ATG5/ATG7 expression, and Beclin-1/Bcl-2 dysregulation. In melanin-feeding HaCaT cells, CoQ_0_ induced melanin degradation by suppressing melanosome gp100 and melanin formation via autophagy. TEM confirmed that CoQ_0_ increased melanosome-engulfing autophagosomes and autolysosomes in melanin-feeding HaCaT cells. Treatment with 3-MA reversed CoQ_0_-mediated melanin degradation in melanin-feeding HaCaT cells. In vivo study showed that CoQ_0_ suppressed endogenous body pigmentation by antimelanogenesis and melanin degradation through autophagy induction in a zebrafish model.

**Conclusions:**

Our results showed that CoQ_0_ exerted antimelanogenesis and melanin degradation by inducing autophagy. CoQ_0_ could be used in skin-whitening formulations as a topical cosmetic application.

**Supplementary Information:**

The online version contains supplementary material available at 10.1186/s12964-024-01537-6.

## Background

Melanogenesis is the synthesis of melanin, a darkly pigmented biopolymer produced by lysosome-like organelles called melanosomes present in melanocytes. Melanogenesis is considered a complex physiological process that involves both melanocytes and keratinocytes. Functionally, melanin is produced from melanocytes and transferred to keratinocytes that surround the melanocytes. The ratio between melanocytes and keratinocytes is approximately 1:36. Therefore, the metabolism (formation and degradation) of melanosomes and melanin determines the skin tone [1]. In addition to melanin metabolism, intrinsic (skin types and genetic background) and extrinsic factors (sunlight exposure levels and pollution) also influence skin color. The most important positive regulator of melanogenesis is melanocortin 1 receptor (MC1R), which is expressed on melanocytes and stimulated by keratinocyte-secreted α-melanocyte stimulating hormone (α-MSH) [2]. Activated MC1R subsequently activates cAMP response element-binding protein (CREB) and microphthalmia-associated transcription factor (MITF), a principal factor regulating melanogenesis [2]. The MITF protein controls the transcription and translation of pigmentary tyrosinase and tyrosinase-related protein-1/-2 (TRP-1/-2) catalytic enzymes, leading to melanin production [3]. Melanin aids in shielding the skin from risky environmental influences (toxic drugs, chemicals, and UV radiation). The most common target in inhibiting pigmentation and melanogenesis pathway is direct inhibition of tyrosinase catalytic activity. Several skin whitening compounds have been used through tyrosinase inhibition [4, 5]. Therefore, most of the commercial skin-whitening products are categorized as tyrosinase inhibitors to lessen hyperpigmentation and preventing cytotoxicity to normal/healthy melanocytes.

Previous studies have documented novel mechanisms and the role of autophagy in depigmenting skin [6]. Defective autophagy mechanisms are associated with various skin pigmentation disorders [7]. Our previous studies confirmed that Vitamin C derivatives (3-O-ethyl ascorbic acid), pterostilbene, and ellagic acid have skin depigmentation efficacy through autophagy induction in melanocytes or keratinocytes [6, 8, 9]. In the cellular microenvironment, autophagy functions as a housekeeping process. Autophagy removes misfolded or aggregated proteins and clears damaged/unnecessary organelles in the cell through a lysosome-dependent mechanism [10]. The key autophagic microtubule-associated protein light chain 3 (LC3) is soluble and widely present in mammalian cells. LC3 exists in cytoplasmic LC3-I and membrane-bound LC3-II (transformed from LC3-I) forms. The conversion of LC3-I to LC3-II is necessary for autophagy. A stable interaction occurs between LC3-II and the autophagosome membrane during autophagy. In the process of autophagy, autophagosomes fuse with lysosomes to form autolysosomes, and intraautophagosomal components are degraded by lysosome hydrolases. Thus, the development of autophagy inducers confers extensive clinical benefit for the treatment of diseases as well as for the regulation of human skin pigmentation by melanocytes and keratinocytes [11].

In Taiwan, *Antrodia camphorata* is locally called Chang-Chih and has been utilized in traditional Chinese medicine. *Antrodia camphorata* was used by aboriginal Taiwanese individuals to cure many illnesses, including hypertension, liver-related disease, itchy skin, abdominal pain, diarrhea, alcohol intoxication, and tumorigenic diseases [12]. Benzoquinone derivatives, quinones, triterpenoids (ergostane and lanostane types), diterpenoids, sesquiterpenoids, benzenoids, lignans, and succinic/maleic acid derivatives have been detected in the culture broths, fruiting bodies, and mycelium of *Antrodia camphorata* [13]. Additionally, *Antrodia camphorata* exhibits anticancer, antioxidant, antiviral, anti-inflammatory, hepatoprotective, antihypertensive, neuroprotective, anti-angiogenesis, and vasorelaxation activities [12].

CoQ_0_, a novel quinone derivative of *Antrodia camphorata*, is the major molecule of CoQ without an isoprenoid side chain. Chemically, CoQ_0_ is known as 2,3-dimethoxy-5-methyl-1,4-benzoquinone [14]. CoQ_0_ biological pharmacology has been reported in vivo and in vitro and in several studies. CoQ_0_ exerts multiple actions including stimulating insulin production in the islets of Langerhans, anti-angiogenic and anti-oxidative defenses, and pharmacological actions against various cancers [14]. However, researchers have not assessed the ability of CoQ_0_ to reduce skin pigmentation. To the best of our knowledge, this investigation is the first to delineate the autophagy mediated depigmenting effects of CoQ_0_ in in vitro (B16F10 and HaCaT cells) and in vivo (zebrafish model).

## Materials and Methods

### Reagents and chemicals

Common cell culture reagents, such as the penicillin‒streptomycin antibody mixture, _L_-glutamine, fetal bovine serum (FBS), and Dulbecco’s modified Eagle’s medium (DMEM), were purchased from Gibco Company (Dublin, Ireland). Coenzyme Q_0_ (CoQ_0_, purity > 99%), 3-[4,5-dimethyl-2-yl]-2,5-diphenyl tetrazolium bromide (MTT), 1-phenyl-2-thiourea (PTU), 3-methyladenine (3-MA), _L_-DOPA, arbutin, and dimethyl sulfoxide (DMSO) were obtained from Sigma-Aldrich Chemical Co. (St. Louis, MO, USA). Antibodies against p-CREB, TRP-1, TRP-2, p-AKT, GAPDH, and β-actin were supplied by Santa Cruz Biotechnology Inc. (Heidelberg, Germany). Additionally, the GeneTex International Corporation (Hsinchu, Taiwan) supplied tyrosinase, MC1R, CREB, MITF, and p-MITF antibodies. In addition, the anti-melanoma glycoprotein 100 (gp100) antibody was obtained from Abcam Inc. (Burlingame, CA, USA). Moreover, LC3-I/II, ERK, p-ERK, JNK, p-JNK, p-PI3K, PI3K, AKT, ATG4B, autophagy-related protein 7 (ATG7), autophagy-related protein 5 (ATG5), Beclin-1, Bcl-2, and histone H3 antibodies were obtained from Cell Signaling Technology (Beverly, MA, USA). Premo™ Autophagy Sensor LC3B-GFP was purchased from ThermoFisher Scientific (Waltham, MA, USA). DAPI was procured from Southern Biotech (Birmingham, AL, USA). Chloroquine (CQ) was obtained from Toronto Research Chemicals (Toronto, Canada). All other general reagents and miscellaneous labware of commercial grade were obtained from Sigma-Aldrich (St. Louis, MO, USA) or Merck & Co., Inc. (Darmstadt, Germany).

### *Antrodia camphorata* fermented broth preparation from submerged culture

*Antrodia camphorata* was collected from Nantou County, Taiwan. All AC specimens used were saved in the CMU repository and named “CMU-AC010”. Dr. Shy-Yuan Hwang from ‘The Endemic Species Research Institute’ in Nantou, Taiwan, characterized the fermented broth prepared from *Antrodia camphorata*. The samples were prepared as previously described [15]. All powdered samples were rendered in DMEM containing 1% FBS (pH 7.4) and were saved at -20 °C. Approximately 2 to 4 batches of fermented *Antrodia camphorata* culture were involved in our experiments. To create a concentrated cultured broth, it was vacuum-sealed and then frozen. From 5 liters, this process produced 46.8 g of dry matter.

### CoQ_0_ isolation from the fermented broths of *Antrodia camphorata* by MPLC and HPLC analysis

*Antrodia camphorata* was eluted by the mobile phase consisting of water and methanol at a flow rate of 40.0 mL/min following a gradient program: 0-40 min, 10-50%; 40-60 min, 50-100% on an RP-18 MPLC glass column (iLOK^TM^, 36.3 x 204 mm; 40-63 μm). UV exposure at 210, 254, and 280 nm was used. Ten fractions (AC-1 to AC-10) were ascertained by using an RP-C18 HPLC column (Cosmosil, 5C18-AR-II, 4.6 × 250 mm; 5 μm). HPLC was performed in an LC-20AT HPLC System (Shimadzu Corporation, Japan) equipped with an SPD-M20A photodiode array detector with a temperature of 25 °C. The gradient elution procedure with mobile phase A (0.05% trifluoroacetic acid in water, *≥*99.0%, Merck, Darmstadt, Germany) and mobile phase B (methanol, *≥*99.0%, Merck, Darmstadt, Germany) was as follows: 0-15 min, 90-60% A; 15-60 min, 60-50% A; 60-70 min, 50-0% A. Ten fractions (AC-1 to AC-10) were generated by dilution in methanol (5.0 mg/mL), filtration through a 0.22 μm membrane filter, and HPLC analysis. Ultraviolet exposure at 280 nm was used.

### Cell culturing and CoQ_0_ stock preparation

Murine melanoma B16F10 cells (Cat # CRL-6475) were purchased from the American Type Culture Collection (ATCC, VA, USA). Human skin keratinocyte HaCaT cells (Cat # 300493) were obtained from Cell Line Services (CLS, Eppelheim, Germany). Complete DMEM supplemented with FBS and antibiotics was used to culture HaCaT and B16F10 cells, as described previously [9]. A 100 mM CoQ_0_ stock concentration was prepared in DMSO and stored at -20 °C until further use.

### MTT (cell viability) assay

Cultured cells (HaCaT or B16F10) were treated with varying concentrations of CoQ_0_ for the given conditions. After treatments, the cells were washed with PBS, and MTT was added to each well (0.5 mg/mL for 2 h). Following the incubation period, solubilization of cytoplasmic formazan crystals was performed using DMSO solution (0.8 mL), and the color intensity that developed was quantified using an ELISA plate reader. The wavelength was set to 570 nm (λ_570_) [6].

### Immunoblotting assay

The immunoblotting assay was conducted to determine the expression of various proteins present in the cells (HaCaT/B16F10) exposed to varying concentrations of CoQ_0_ for different durations. The protocols for harvesting protein samples and the immunoblotting assay were described previously [16]. Densitometry analyses were conducted to measure the protein expression levels, and commercial software (AlphaEase, Genetic Technology Inc., Miami, FL, USA) was used for data acquisition. β-Actin, histone H3, and GAPDH served as loading control proteins.

### Immunofluorescence staining

Immunofluorescence staining was performed as described previously [16]. Briefly, 3-MA (1 mM, 1 h)-pretreated or nontreated HaCaT cells were subsequently exposed to various concentrations of CoQ_0_ (0-5 μM for 24 h). After incubation, the cells were subjected to staining using different primary (anti-LC3B, anti-gp100, anti-tyrosinase, and anti-MITF) and fluorescein isothiocyanate (FITC)-conjugated (488 nm) secondary antibodies. DAPI was used for nuclear (counter)staining. After staining, the distribution of antibodies in the stained cells was examined under a fluorescence microscope.

### LC3 transfection studies

Cells (B16F10/HaCaT) were grown in 6-well plates and used for transfection when they reached 50% confluence. A commercial transfection kit (Lipofectamine RNAiMAX from Invitrogen, Carlsbad, CA, USA) was used for transfection. The procedure used to transfect LC3 plasmids was described in a previous study [6].

### Estimation of the melanin content

Cells (B16F10/HaCaT) were cultured in a 10-cm dish to 50% confluence. Cells were collected and rinsed with PBS. At the end of the treatments, the cells were rinsed with PBS again and harvested to measure the melanin content. This procedure was explained in detail in a previous study [17].

### GFP-LC3 puncta formation

The GFP-LC3 fusion protein was used to detect autophagosomes (cellular GFP-LC3 puncta) in the cells. The Premo™ Autophagy Sensor LC3B-GFP Kit from Thermo Fisher Scientific (Waltham, MA, USA) was used according to the manufacturer’s protocol [18]. After incubation, the culture medium was replaced with fresh medium, and the cells were subjected to CoQ_0_ treatment for the indicated durations. After treatments, the cells were washed with PBS, and cellular GFP-LC3 puncta were observed under a laser scanning confocal microscope.

### Detection of acidic vesicular organelles (AVOs) using acridine orange staining

Acridine orange (AO) is a fluorescence-based cationic dye that is also cell membrane-permeable and serves as a marker dye to detect AVOs that accumulate within cells. Based on this proportionality principle, the level of AVOs present in the cells was categorized as high (red), intermediate (yellow), and low (green). The protocol for AO staining was followed as described in a previous study [6]. After treatments, 1 μg/mL AO stain prepared in PBS + 5% FBS was applied for approximately 15 min, and the cells were observed under a fluorescence microscope to detect AVOs. For the quantification of fluorescence, Olympus Softimage Solution software (Olympus Imaging America, Inc., PA, USA) was applied.

### Tyrosinase activity

Tyrosinase activity was measured in B16F10 cells. The method used to determine tyrosinase activity was described in a recent study [9]. The following formula was applied to measure cellular tyrosinase activity:

$$\mathrm{Tyrosinase\ activity }\left(\mathrm{\%}\right)= \frac{\uplambda 475\mathrm{\ of\ sample}}{\uplambda 475\mathrm{\ of\ control}}\times 100$$

### Melanin-feeding HaCaT cells

The Sigma-Aldrich Chemical Company supplied synthetic melanin (St. Louis, USA). Melanin (25 ng/mL) was fed to HaCaT cells for 24 h before they were given CoQ_0_ for the indicated time [16].

### Transmission electron microscopy (TEM)

TEM was used to observe the key cellular events in B16F10 and melanin-feeding HaCaT cells. After the treatments were applied, cells were fixed and prepared for TEM observation (Tecnai 12, FEI, Hillsboro, Oregon, USA) as described previously [6].

### In vivo zebrafish studies

The depigmenting effect of CoQ_0_ in vivo was assessed in the zebrafish model. These in vivo experiments were approved by the China Medical University Institutional Animal Care and Use Committee (IACUC). The protocol used to measure this parameter was described in a previous report [6]. Changes in zebrafish viability due to exposure to CoQ_0_, the heartbeat rate, and endogenous pigmentation were monitored through a Z16 stereomicroscope (Leica Microsystems, Ernst-Leitz-Strasse, Germany). Image-Pro Plus software (Media Cybernetics, Inc., Rockville, USA) was used to analyze the data. The methodology used to measure melanin production in zebrafish was described previously [6].

### Analyses of LC3B and tyrosinase protein expression in zebrafish embryos

Zebrafish embryos (9 hpf) were treated with CoQ_0_ (0-10 μM) or vehicle (0.1% DMSO) for up to 72 hpf. After incubation, proteins were extracted from the embryos using a previously reported method [6]. The extracted proteins were subjected to Western blotting to measure LC3B and tyrosinase protein expression. The immunoblotting procedure is described earlier in this section.

## Statistical analysis

Analysis of variance (ANOVA) and Dunnett’s test were performed for pairwise comparisons among the control and test groups. SigmaPlot 10.0 statistical software was employed for the analyses. The data are reported as fold or 100% changes.

## Results

### Isolation and analysis of CoQ_0_ derived from ***Antrodia camphorata*** by MPLC and HPLC analysis

**Fig. 1:**
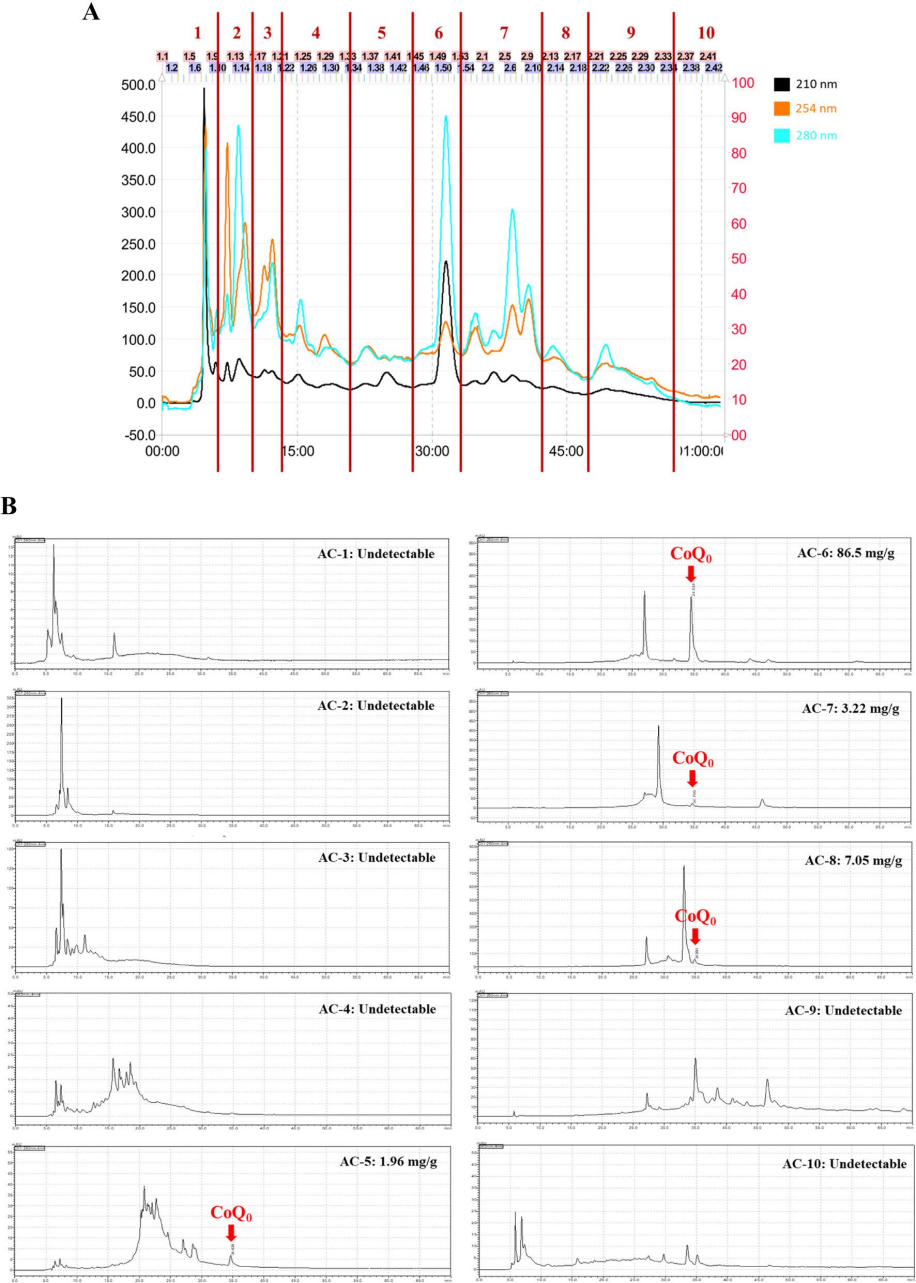
CoQ_0_ isolation from *Antrodia camphorata* by MPLC and HPLC analysis. **A** *Antrodia camphorata* was eluted by a mobile phase consisting of water and methanol by using an RP-18 MPLC glass column. **B** Ten fractions (AC-1 to AC-10) were ascertained by using an RP-C18 HPLC column. A series of standard solutions (CoQ_0_) were created by diluting the stock solution, and they were utilized to calculate the CoQ_0_ concentration of the AC-1 to AC-10 fractions. The final CoQ_0_ product was dissolved in 0.1% DMSO

**Fig. 2:**
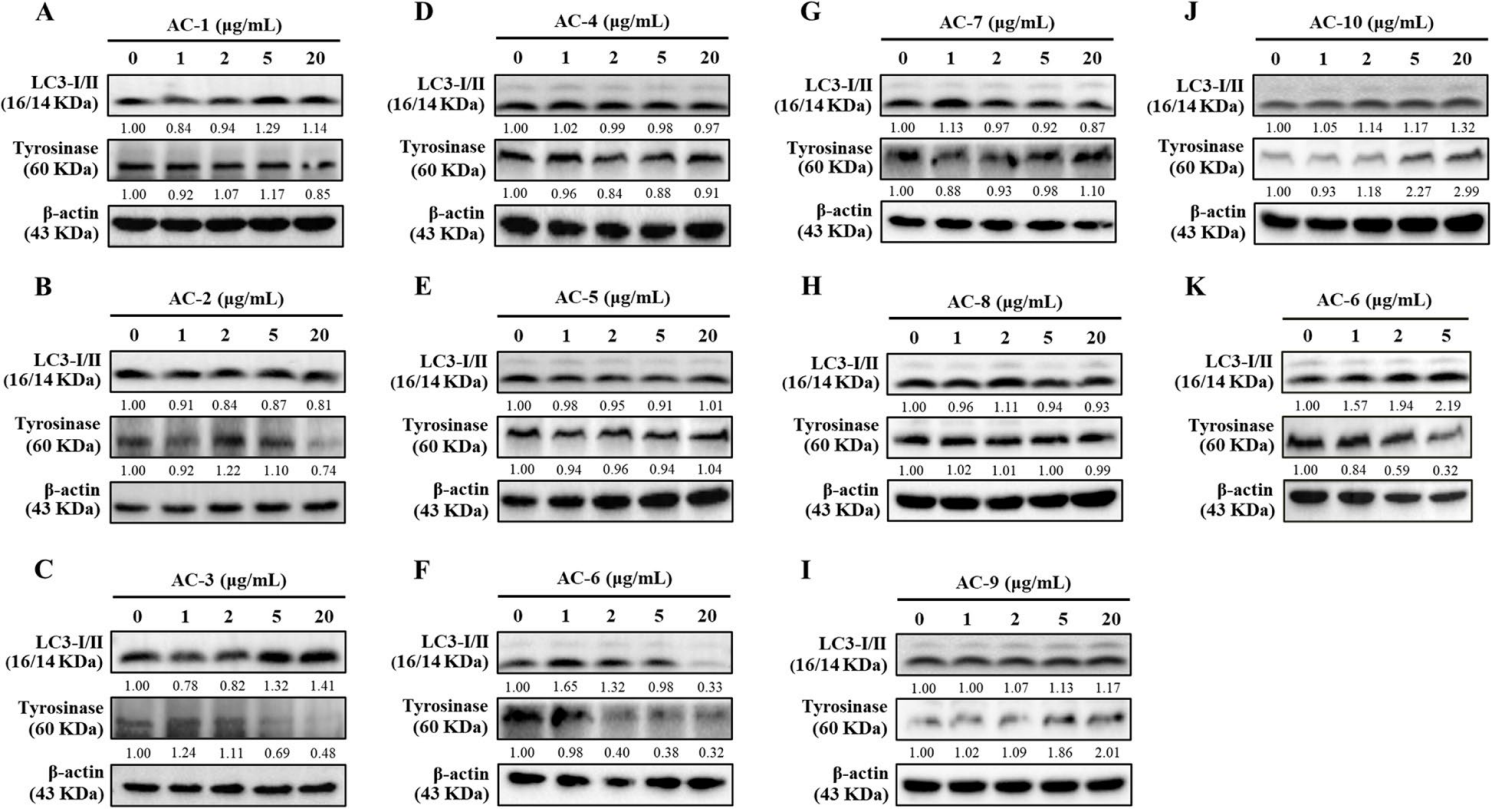
AC-6 upregulated LC3-I/II and inhibited tyrosinase expression in melanoma B16F10 cells. **A-K** B16F10 cells were treated with AC-1 to AC-10 (0-20 μg/mL) or AC-6 (0-5 μg/mL) for 24 h. The expression of LC3-I/II and tyrosinase was evaluated by immunoblotting

MPLC profiling was performed on the fermented culture broths of *Antrodia camphorata*, which were separated into ten fractions (AC-1 to AC-10, Fig. 1A). The CoQ_0_ concentrations of the AC-1 to AC-10 fractions derived from *Antrodia camphorata* are shown in Fig. 1B. HPLC analysis revealed that the highest CoQ_0_ concentration was 86.6 mg/g in the AC-6 fraction **(**Fig. 1B**)**. The effect of autophagy on the antimelanogenesis of AC-1 to AC-10 fractions (0-20 μg/mL, Fig. 2A-J) was subsequently assessed using immunoblotting in melanoma B16F10 cells. Interestingly, the AC-6 fraction remarkably upregulated the expression of the autophagy marker LC3-II and remarkably downregulated the expression of key melanogenesis-related tyrosinase in B16F10 cells (Fig. 2F and K). The results suggested that CoQ_0_ derived from *Antrodia camphorata* might be an effective compound for use in depigmenting cosmetics. Consequently, we aimed to delineate the depigmenting mechanisms of CoQ_0_, a novel quinone derivative of *Antrodia camphorata*, by inducing autophagy in melanoma B16F10 and keratinocyte HaCaT cells.

### CoQ_0_ suppressed melanogenesis in unstimulated or α-MSH-stimulated B16F10 cells

**Fig. 3:**
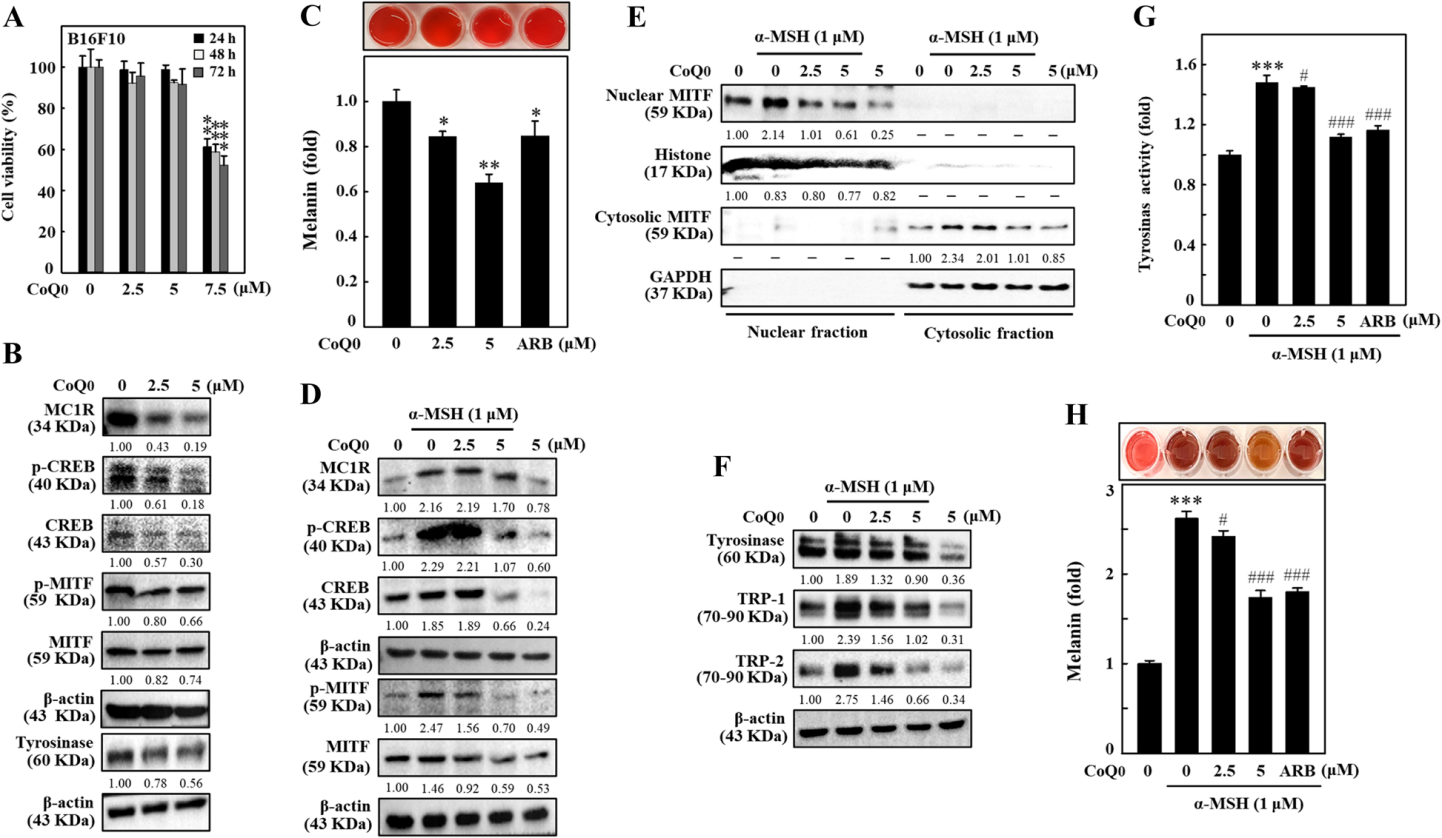
CoQ_0_ suppressed melanogenesis in α-MSH-stimulated B16F10 cells. **A** Cells were incubated with CoQ_0_ (0-7.5 μM) for 24, 48, or 72 h, and an MTT assay was conducted to determine cell viability. **B** Cells were treated with CoQ_0_ (0-5 μM) for the indicated durations, and the levels of MC1R (5 min), p-CREB (1.5 h), CREB (1.5 h), p-MITF (4 h), MITF (4 h), and tyrosinase (24 h) were measured using immunoblotting. **C** Cells were treated with CoQ_0_ (0-5 μM, 72 h), and melanin formation was determined as described in the Methods section. **D**, **F** Cells were first treated with CoQ_0_ (0-5 μM), followed by stimulation with α-MSH (1 μM) for the indicated time to measure (**D**) MC1R (5 min), p-CREB (1.5 h), CREB (1.5 h), p-MITF (4 h), and MITF (4 h) levels; (**E**) nuclear and cytosolic MITF (4 h) levels; and (**F**) tyrosinase (24 h), TRP-1 (24 h), and TRP-2 (24 h) levels using immunoblotting. **G**, **H** Tyrosinase activity was determined after 24 h, and melanin levels were measured after 72 h, as described in the Methods section. Arbutin (ARB, 200 μM) was used as a positive control. The results are the mean ± SD (*n*=3). *p < 0.05; **p< 0.01; ***p < 0.001 compared with untreated cells. ^#^*p* < 0.05; ^###^*p* < 0.001 compared with CoQ_0_-treated cells

We determined the cytotoxic concentration of CoQ_0_ (0-7.5 μM for 24, 48, or 72 h) in B16F10 cells. MTT results demonstrated that compared to the untreated control, B16F10 cell viability was significantly decreased after treatment with 5 μM CoQ_0_, and this repression was also aggravated with increasing CoQ_0_ incubation time (Fig. 3A). Therefore, ≤ 5 μM CoQ_0_ was considered a nontoxic or subcytotoxic CoQ_0_ concentration for further in vitro experiments in this investigation (Fig. 3A). In B16F10 cells, we first examined the efficacy of CoQ_0_ with respect to the protein expression involved in melanogenesis. The immunoblotting data revealed that CoQ_0_ dose-dependently decreased the levels of the MC1R, p-CREB, CREB, p-MITF, MITF, and tyrosinase proteins (Fig. 3B). Additionally, melanin formation was dose-dependently downregulated by CoQ_0_ (Fig. 3C). MC1R expressed on melanocytes is a critical positive modulator of melanogenesis which is instigated by keratinocyte-released α-MSH [19]. The MC1R transduces the upstream signal to generate cAMP via adenyl cyclase and activates the transcription factors CREB and MITF which enhance the expression of tyrosinase, TRP-1, and TRP-2, leading to increased melanin production [20]. We subsequently evaluated the antimelanogenic effect of CoQ_0_ on α-MSH (1 μM)-stimulated B16F10 cells. Western blot data indicated that α-MSH stimulation upregulated MC1R, p-CREB, CREB, p-MITF, and MITF levels in B16F10 cells (Fig. 3D). In contrast, CoQ_0_ dose-dependently decreased the levels of MC1R, p-CREB, CREB, p-MITF, and MITF proteins in α-MSH-stimulated B16F10 cells (Fig. 3D). Furthermore, CoQ_0_ (2.5 and 5 μM) dose-dependently enhanced the nuclear localization of the MITF protein in α-MSH-stimulated B16F10 cells (Fig. 3E). Moreover, α-MSH stimulation upregulated tyrosinase, TRP-1, and TRP-2 levels in B16F10 cells. However, tyrosinase, TRP-1, and TRP-2 expression decreased after treatment with increasing CoQ_0_ concentrations in α-MSH-stimulated B16F10 cells (Fig. 3F). Interestingly, consistent with the immunoblotting data, tyrosinase enzyme activity and melanin levels were also markedly decreased in CoQ_0_-treated B16F10 cells stimulated with α-MSH (Fig. 3G and H). Based on these data, the mechanism by which CoQ_0_ suppressed MC1R-CREB-mediated MITF expression and led to the downregulation of tyrosinase and TRP-1/-2 expression and melanin synthesis was elucidated, suggesting that CoQ_0_ suppressed melanogenesis in unstimulated or α-MSH-stimulated B16F10 cells.

### CoQ_0_ inhibited MITF nuclear translocation through the ERK, JNK and PI_3_K/AKT pathways in B16F10 cells

**Fig. 4:**
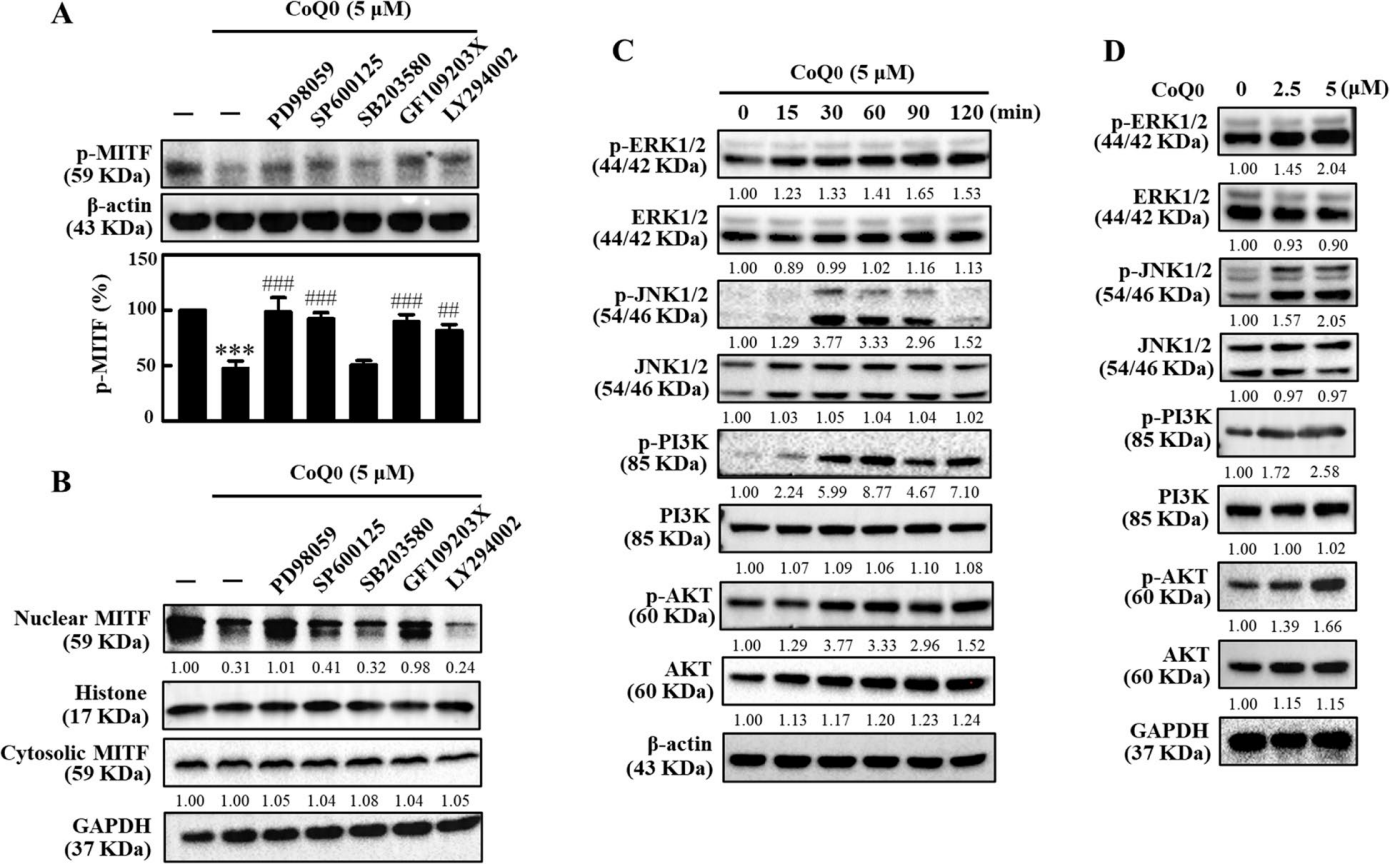
CoQ_0_ suppressed MITF nuclear translocation in B16F10 cells through the ERK, JNK, and PI3K/AKT signaling pathways. **A**, **B** Cells were first treated with inhibitors of ERK (PD98059, 30 μM), JNK (SP600125, 25 μM), p38 (SB203580, 20 μM), PKC (GF109203X, 2.5 μM), or PI3K/AKT (LY294002, 30 μM) for 1 h followed by CoQ_0_ (5 μM, 4 h). Levels of p-MITF, and nuclear/cytosolic MITF were determined using immunoblotting. **C** Cells were treated with CoQ_0_ (5 μM, 0-120 min). An immunoblotting assay was performed to determine the levels of the p-ERK1/2, ERK1/2, p-JNK1/2, JNK1/2, p-PI3K, PI3K, p-AKT, and AKT proteins. **D** Cells were treated with CoQ_0_ (0-5 μM) for the indicated time, and immunoblotting was performed to determine the levels of the p-ERK1/2 (90 min), ERK1/2 (90 min), p-JNK1/2 (30 min), JNK1/2 (30 min), p-PI3K (60 min), PI3K (60 min), p-AKT (60 min), and AKT (60 min) proteins. The results are the mean ± SD (*n*=3). ***p < 0.001 compared with untreated cells. ^###^*p* < 0.001 compared with CoQ_0_-treated cells

We next investigated the roles of different signaling pathways in CoQ_0_-mediated inhibition of MITF nuclear translocation in B16F10 cells [3]. Various pharmacological inhibitors were used for this experiment. The immunoblot results showed that the ERK (PD98059), JNK (SP600125), or PI3K/AKT (LY294002) inhibitors remarkably reversed the CoQ_0_ (5 μM)-induced decrease in p-MITF and nuclear MITF protein levels, suggesting that the ERK, JNK, and PI3K/AKT pathways play crucial roles (Fig. 4A and B). Moreover, CoQ_0_ (5 μM) increased p-ERK, p-JNK, and p-PI3K/AKT in a time- and dose-dependent manner (Fig. 4C and D). This result confirmed that CoQ_0_ suppressed MITF nuclear translocation in B16F10 cells via the ERK, JNK, and PI3K/AKT pathways.

### CoQ_0_ enhanced LC3-I/II accumulation and autophagic flux in B16F10 cells

**Fig. 5:**
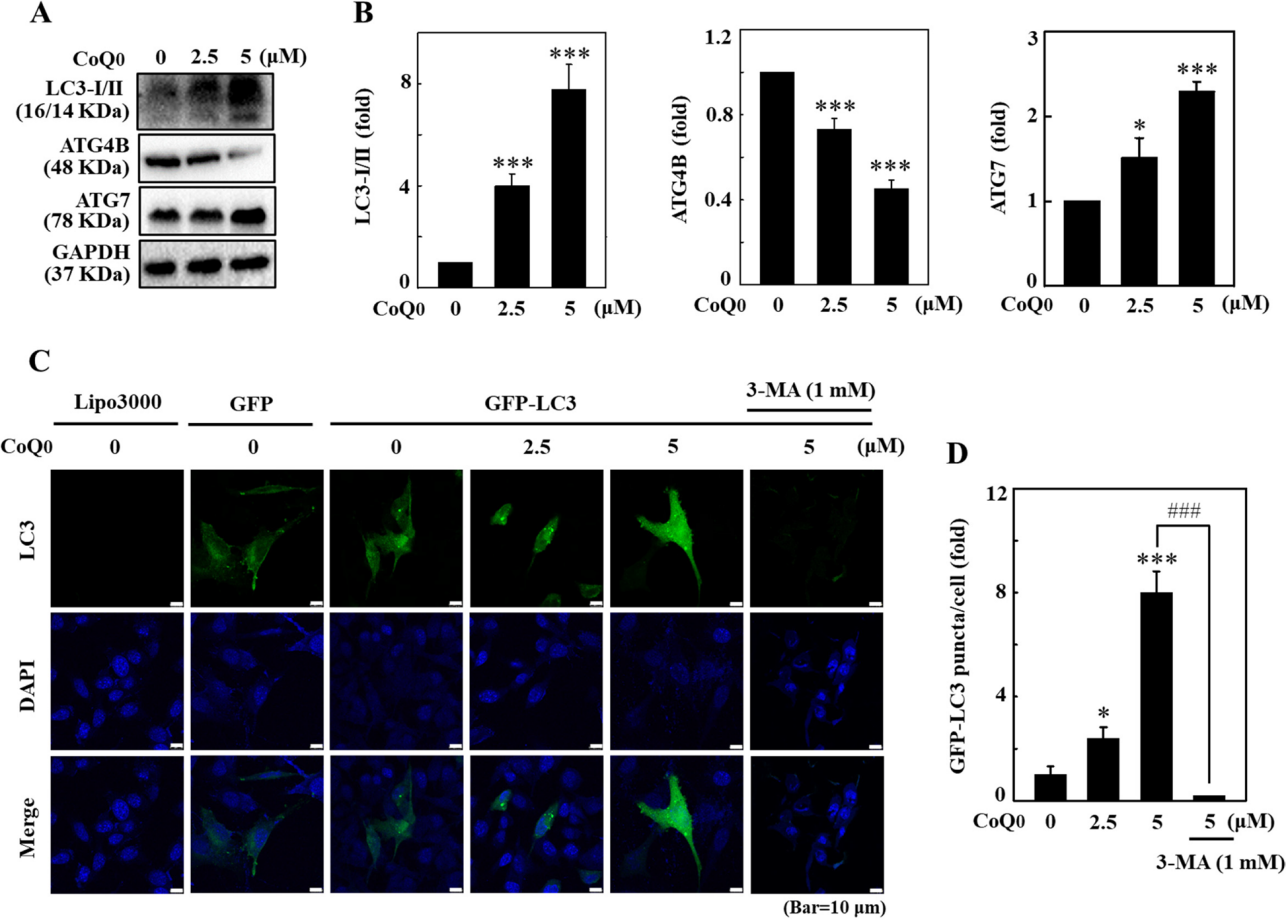
CoQ_0_ induced autophagic flux in B16F10 cells. **A**, **B** Cells were treated with CoQ_0_ (0-5 μM, 24 h). After incubation, the cells were subjected to an immunoblotting assay to determine LC3-I/II, ATG4B, and ATG7 protein levels. **C**, **D** The GFP-LC3 expression vector was transfected into the cells, followed by treatment with CoQ_0_ (0-5 μM, 24 h). GFP-LC3 puncta induced by CoQ_0_ were observed using a confocal microscope. The quantification of cells developing GFP-LC3 puncta is presented in a histogram. The results are the mean ± SD (*n*=3). *p < 0.05; ***p < 0.001 compared with untreated cells. ^###^*p* < 0.001 compared with CoQ_0_-treated cells

In B16F10 cells, the differential expression patterns of an autophagy marker (LC3-I/II) and its associated proteins were determined after treatment with CoQ_0_. The immunoblotting data suggested that in the presence of CoQ_0_ (0-5 μM for 24 h), LC3-I/II expression was significantly upregulated in B16F10 cells (Fig. 5A and B). Autophagy-related 4B cysteine peptidase (ATG4B) negatively regulates autophagy and represses autophagy by eliminating lipid conjugates from LC3 (LC3-II). Therefore, ATG4B serves as a negative regulator of autophagy [21]. Hence, the results confirmed that ATG4B expression was significantly downregulated by CoQ_0_ treatment in B16F10 cells (Fig. 5A and B) and that ATG7 functions as an E-1 enzyme at the beginning of autophagy for ubiquitin-like proteins, including ATG12 and ATG8 [22]. During the initiation of autophagy, ATG7 acts as a critical regulator of autophagosome assembly [22]. ATG7 expression was significantly upregulated by CoQ_0_ treatment in B16F10 cells (Fig. 5A and B). These results suggested that CoQ_0_ enhanced autophagy in B16F10 cells.

### CoQ_0_ increased autophagosome GFP-LC3 puncta in B16F10 cells

GFP-LC3 was transiently transfected into B16F10 cells to further determine whether CoQ_0_ stimulated the development of autophagosomes using confocal microscopy. The data confirmed that CoQ_0_ increased autophagosome formation, as inferred by the increased number of green LC3 puncta in cytoplasmic B16F10 cells (Fig. 5C and D). Interestingly, this result was reversed (significantly downregulated) by the autophagy inhibitor 3-MA, suggesting that autophagosome GFP-LC3 formation was facilitated in CoQ_0_-treated B16F10 cells (Fig. 5C and D).

### CoQ_0_ increased AVO formation in B16F10 cells

**Fig. 6:**
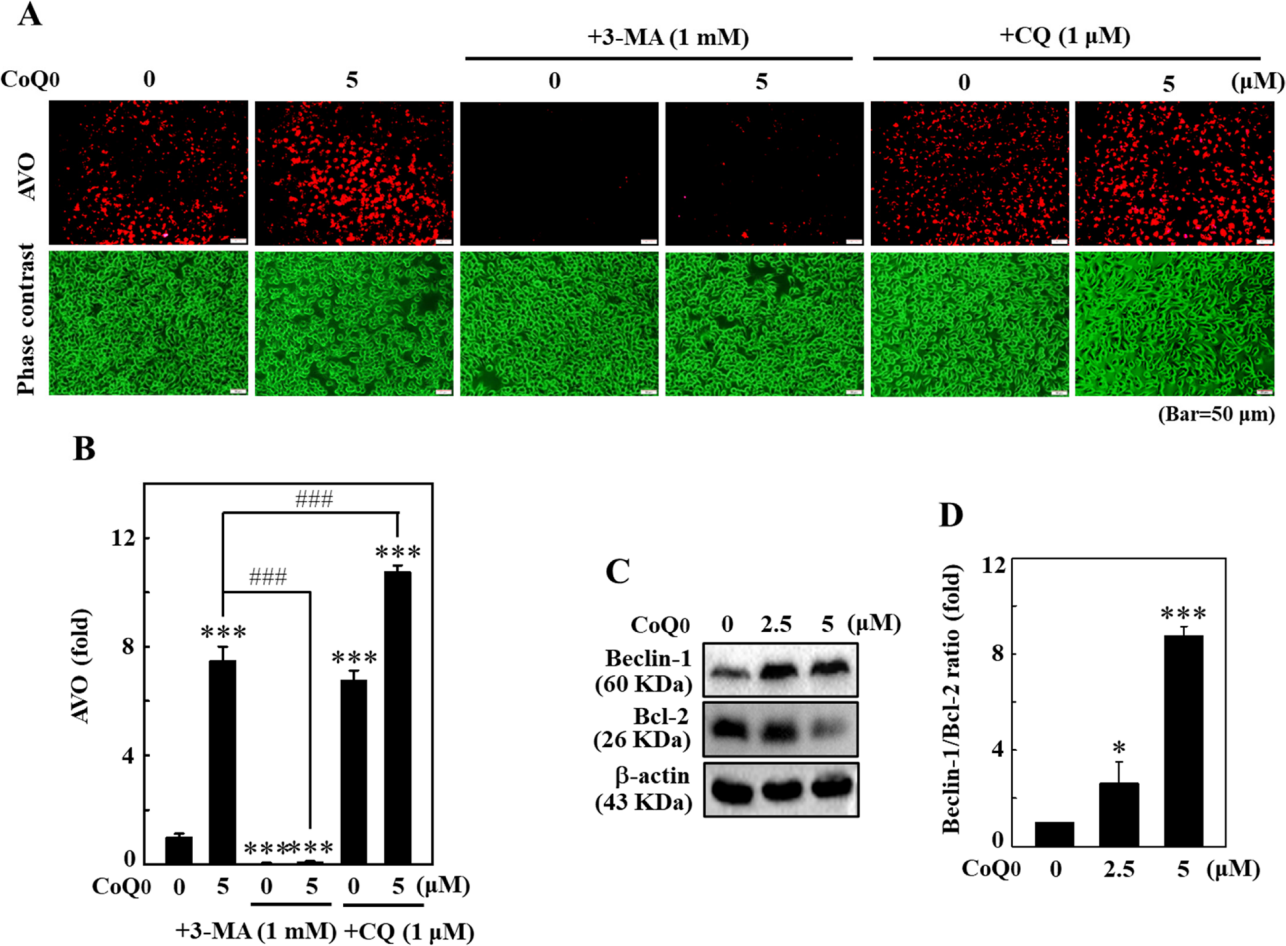
CoQ_0_ increased AVO formation and dysregulated the Beclin-1/Bcl-2 ratio in B16F10 cells. **A**, **B** Cells were first treated with 3-MA (1 mM, 1 h) or CQ (1 μM, 1 h) and then with CoQ_0_ (0 or 5 μM, 24 h). A fluorescence microscope was used to visualize the formation of intracellular AVOs (under a red filter). The intensity of red fluorescence is proportional to the AVO number. **C**, **D** Cells were treated with CoQ_0_ (0-5 μM, 24 h). The immunoblotting assay was performed to determine Beclin-1 and Bcl-2 protein levels, and the data are presented as the ratio of Beclin-1/Bcl-2. The results are the mean ± SD (*n*=3). *p < 0.05; ***p < 0.001 compared with untreated cells. ^###^*p* < 0.001 compared with CoQ_0_-treated cells

The autophagy-inducing efficacy of CoQ_0_ (0-5 μM for 24 h) was further analyzed by measuring AVO formation. AVO levels were measured by performing AO staining. AVOs significantly accumulated in CoQ_0_-treated B16F10 cells (Fig. 6A and B). Moreover, 3-MA (an early autophagy inhibitor) and CQ (a late autophagy inhibitor) were used to evaluate the interconnection with respect to CoQ_0_-induced AVO accumulation. Pretreatment with 3-MA (1 mM) suppressed this AVO accumulation; however, pretreatment with CQ (1 μM) enhanced the effect in CoQ_0_-treated B16F10 cells (Fig. 6A and B). Furthermore, B16F10 cells treated with 3-MA or CQ alone displayed a significant decrease or increase in AVO formation, respectively (Fig. 6A and B).

### CoQ_0_ dysregulated the Beclin-1/Bcl-2 ratio in B16F10 cells

The coordinating role of autophagic Beclin-1 and anti-autophagic Bcl-2 proteins determines autophagy flux in cells [23]. The effect of CoQ_0_ (0-5 μM for 24 h) on Beclin-1 and Bcl-2 expression was evaluated using immunoblotting. CoQ_0_ treatment significantly upregulated Beclin-1 expression but downregulated Bcl-2 expression, leading to a shift in the Beclin-1/Bcl-2 ratio toward favoring autophagy in B16F10 cells (Fig. 6C and D). These results defined CoQ_0_-induced autophagy in B16F10 cells.

### CoQ_0_ repressed p-MITF and tyrosinase expression through autophagy in B16F10 cells

**Fig. 7:**
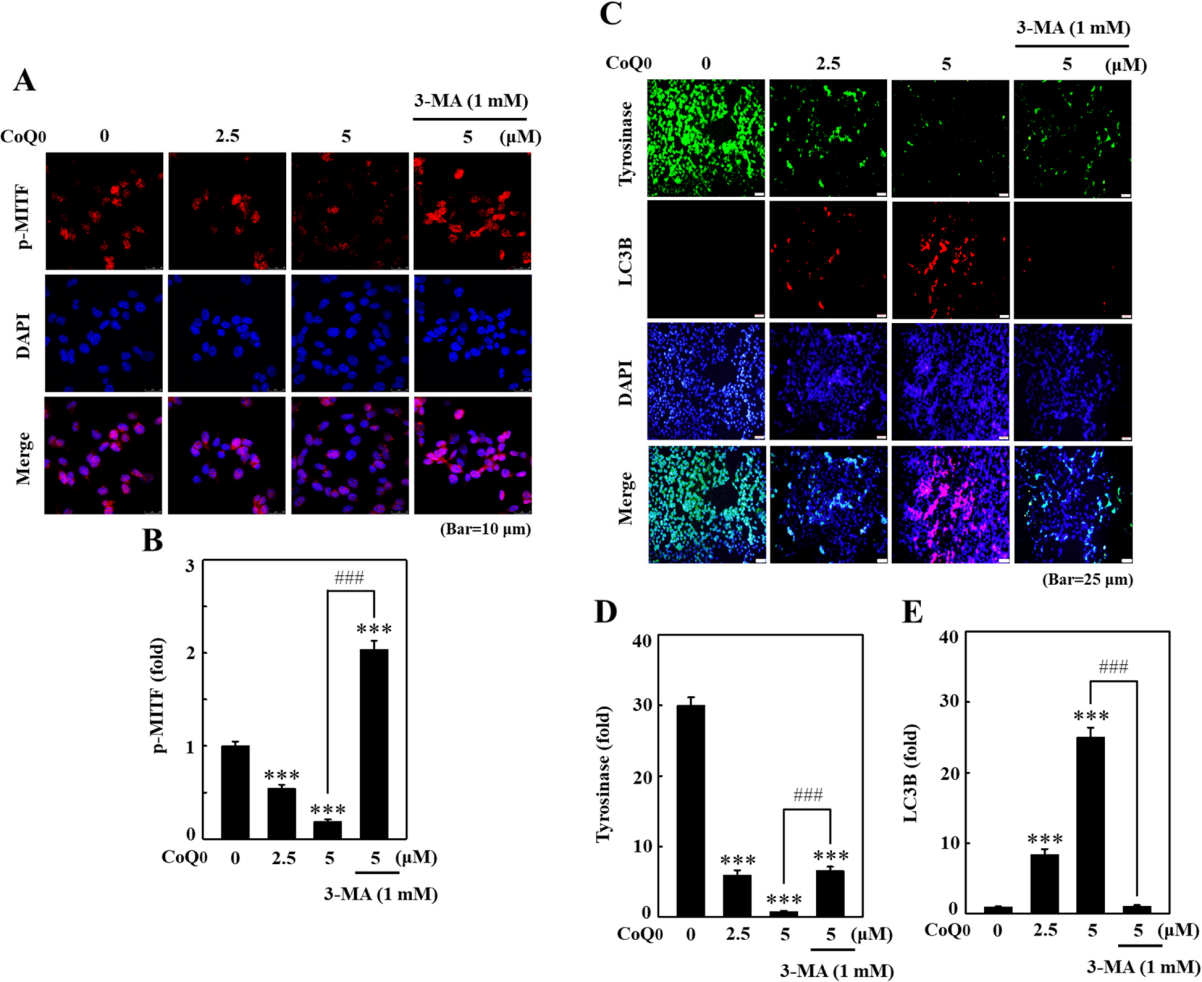
CoQ_0_ suppressed p-MITF and tyrosinase expression through autophagy in B16F10 cells. Cells were pretreated with or without 3-MA (1 mM, 1 h), followed by CoQ_0_ (0-5 μM) for 4 h (**A**, **B**) or 24 h (**C**, **E**). At the end of the treatment, the p-MITF (**A**, **B**), tyrosinase, and LC3B (**C, E**) levels were measured using immunofluorescence staining. The results are the mean ± SD (*n*=3). ***p < 0.001 compared with untreated cells. ^###^*p* < 0.001 compared with CoQ_0_-treated cells

The effect of autophagy on the antimelanogenesis effect of CoQ_0_ was subsequently assessed. For this experiment, 3-MA was administered to determine the key melanogenesis-associated tyrosinase and p-MITF levels using immunofluorescence staining in B16F10 cells. CoQ_0_ (0-5 μM for 24 h) mediated a decrease in p-MITF expression, and this effect was also reversed by 3-MA (Fig. 7A and B). Furthermore, CoQ_0_ significantly reduced tyrosinase levels, whereas LC3B levels were significantly upregulated in B16F10 cells (Fig. 7C, D and E). Notably, CoQ_0_-induced inhibition of tyrosinase expression was reversed by pretreatment with 3-MA (Fig. 7C, D and E). The data demonstrated that autophagy plays a crucial role in the antimelanogenesis effect of CoQ_0_ in B16F10 cells.

### CoQ_0_ inhibited tyrosinase expression/activity and melanin levels by inducing autophagy in α‐MSH-stimulated B16F10 cells

**Fig. 8:**
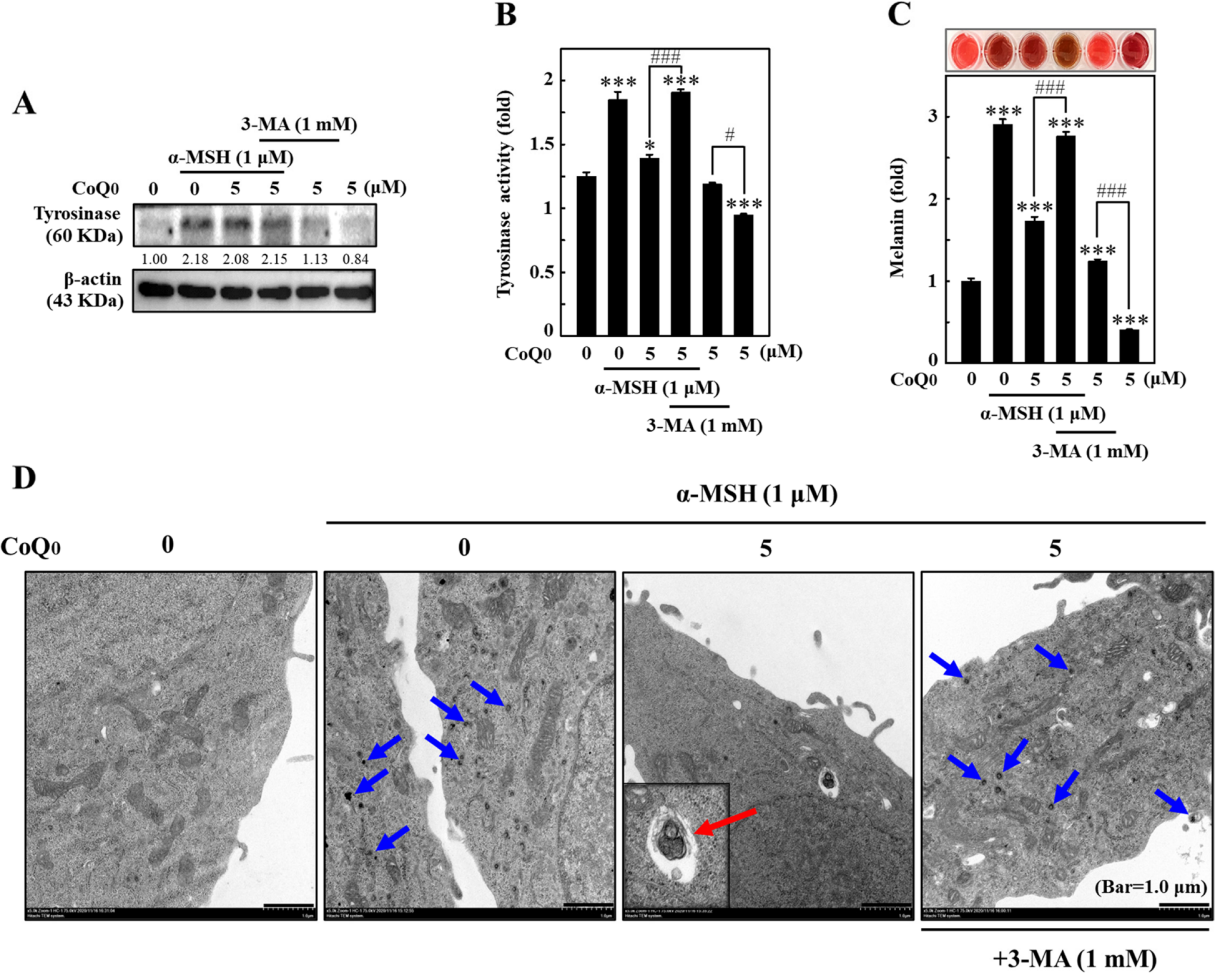
CoQ_0_ inhibited melanogenesis by inducing autophagy in α‐MSH-stimulated B16F10 cells.** A-C** Cells were treated with CoQ_0_ (0 or 5 μM) in the absence or presence of 3-MA (1 mM, 1 h) followed by stimulation with α‐MSH (1 μM) for 24 h (**A**, **B**) or 72 h (**C**). **A** An immunoblotting assay was applied to measure the tyrosinase expression. **B** Tyrosinase enzyme activity was measured as described in Materials and Methods. **C** Intracellular melanin levels were quantified. The results are the mean ± SD (*n*=3). *p < 0.05; ***p < 0.001 compared with untreated cells. ^#^*p* < 0.05; ^###^*p* < 0.001 compared with CoQ_0_-treated cells. **D** Cells were treated with or without 3-MA (1 mM, 1 h) followed by treatment with CoQ_0_ (0 or 5 μM) and stimulation with α‐MSH (1 μM, 24 h). Melanosome-engulfing autophagosomes within cells were observed under TEM. The blue and red arrows indicate melanosome-engulfing autophagosomes, and autolysosomes formation, respectively.

The effects of CoQ_0_ and/or 3-MA on tyrosinase activity/expression and melanin production were measured in α-MSH-stimulated B16F10 cells. Increased tyrosinase expression/activity (Fig. 8A and B) and melanin levels (Fig. 8C) were observed in α-MSH-stimulated B16F10 cells. However, CoQ_0_ (0-5 μM for 24 h) treatment substantially reduced tyrosinase expression/activity and melanin levels in α‐MSH-stimulated B16F10 cells (Fig. 8A, B and C). Moreover, in the presence of 3-MA (1 mM), the attenuating effects of CoQ_0_ were reversed in α-MSH-stimulated B16F10 cells (Fig. 8A, B and C), confirming that autophagy was directed to inhibiting melanogenesis in α-MSH-stimulated B16F10 cells.

### CoQ_0_ triggered melanosome-engulfing autophagosome and autolysosome formation in α-MSH-stimulated B16F10 cells

The effect of CoQ_0_ and/or 3-MA on α-MSH-stimulated melanosome-engulfing autophagosomes was tested using TEM in B16F10 cells. The results revealed that melanosomes formed in α-MSH-stimulated B16F10 cells; nevertheless, CoQ_0_ (0-5 μM for 24 h)-treated cells triggered melanosome-engulfing autophagosome and autolysosome formation (Fig. 8D). Remarkably, these effects were reversed in 3-MA (1 mM)-pretreated B16F10 cells (Fig. 8D). Therefore, the results suggested that the antimelanogenesis effect of CoQ_0_ was facilitated by inducing autophagy in α-MSH-stimulated B16F10 cells.

### LC3 silencing suppressed CoQ_0_-triggered antimelanogenesis in B16F10 cells

**Fig. 9:**
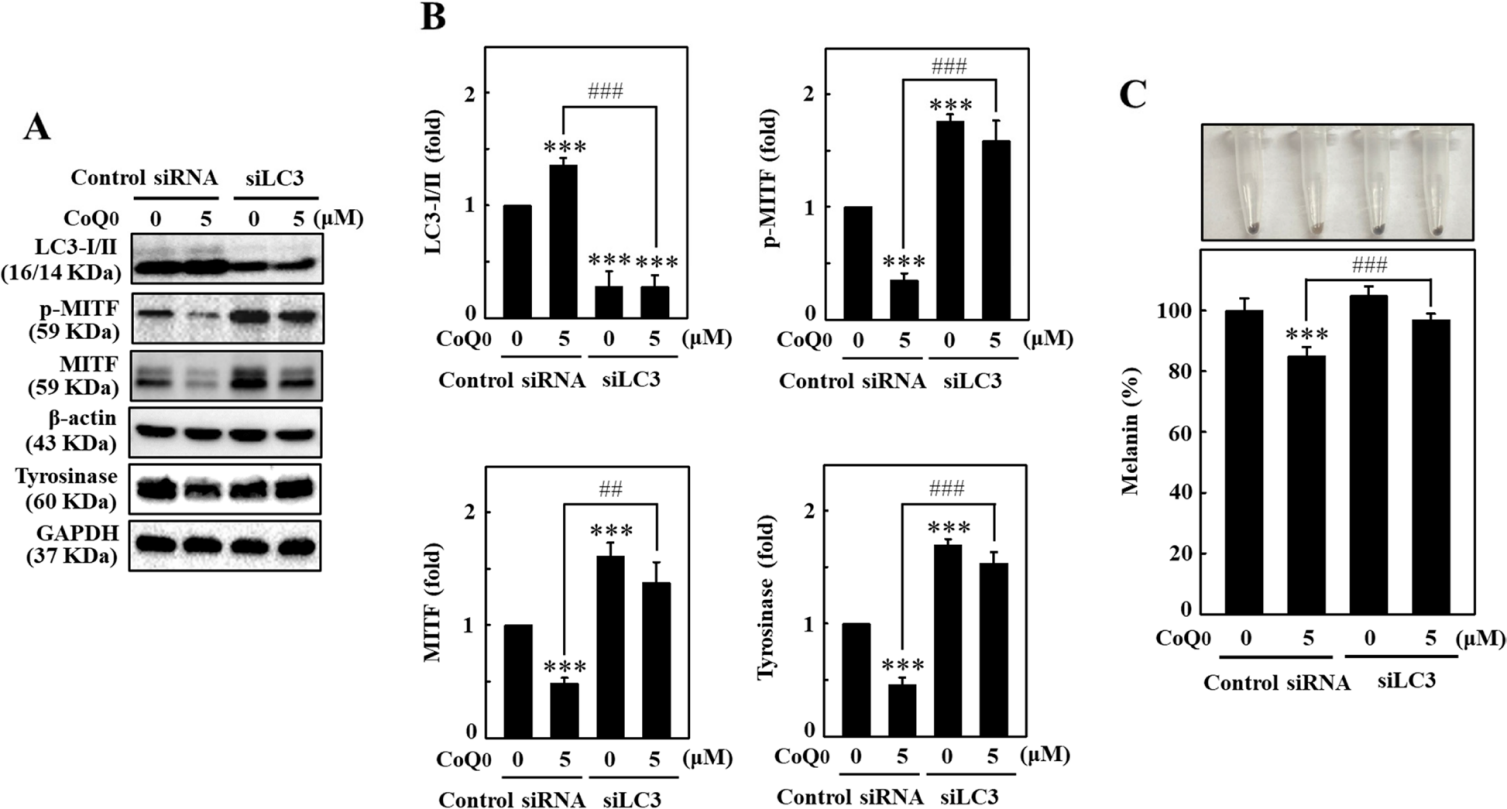
Silencing LC3 diminished CoQ_0_-induced antimelanogenesis in B16F10 cells. **A**, **B** Cells transfected with the control siRNA and siLC3 were treated with CoQ_0_ (0 or 5 μM) for the indicated duration to determine the levels of LC3B (24 h), p-MITF (4 h), MITF (4 h), and tyrosinase (24 h) proteins. The proteins were extracted after various treatments and subjected to immunoblotting. **C** Transfected B16F10 cells were treated with CoQ_0_ (0 or 5 μM, 24 h), followed by the measurement of intracellular melanin levels. The results are the mean ± SD (n=3). **p < 0.01; ***p < 0.001 compared with untreated siRNA-transfected cells. ^##^*p* < 0.01; ^###^*p* < 0.001 compared with CoQ_0_-treated siRNA-transfected cells.

LC3 knockdown cells were used to further examine the importance of CoQ_0_-triggered autophagy in antimelanogenesis. Immunoblots showed significantly decreased LC3-II expression in siLC3-transfected cells after CoQ_0_ (5 μM for 24 h) treatment, confirming that LC3 was successfully knocked down in B16F10 cells (Fig. 9A and B). The levels of p-MITF, MITF, and tyrosinase were decreased in the CoQ_0_-treated control siRNA-transfected cells (Fig. 9A and B). Nevertheless, significant increases in p-MITF, MITF, and tyrosinase expression were detected in CoQ_0_-treated siLC3-transfected cells (Fig. 9A and B). The data also confirmed that CoQ_0_ reduced melanin levels in control siRNA-transfected cells; however, siLC3-transfected cells treated with CoQ_0_ displayed a significant increase in melanin formation (Fig. 9C). These results indicated that autophagy (LC3 function) plays a pivotal role in CoQ_0_-inhibited melanogenesis in B16F10 cells.

### CoQ_0_ modulated autophagy-associated proteins in favor of autophagy in HaCaT cells

**Fig. 10:**
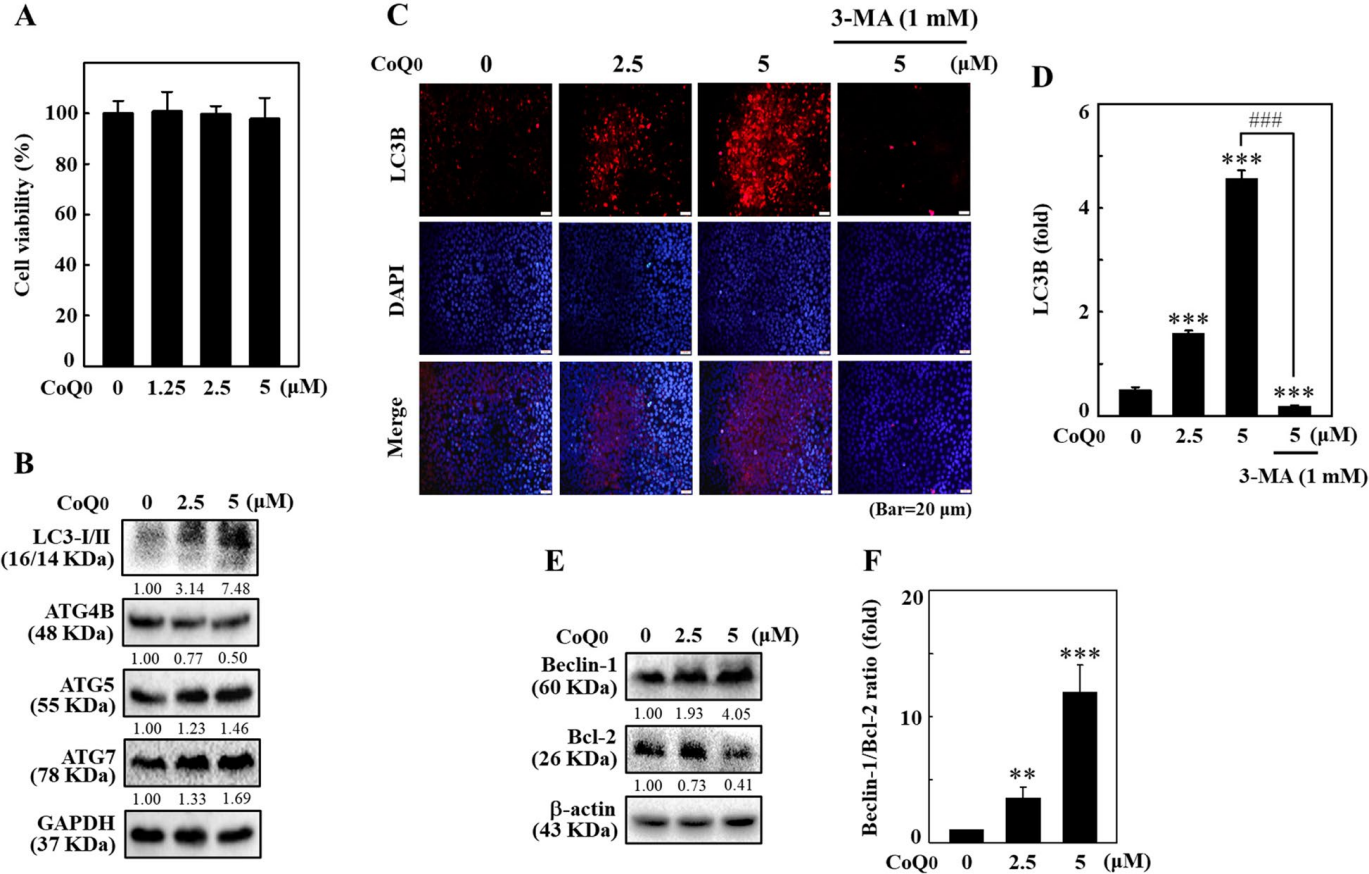
CoQ_0_ modulated autophagy-associated proteins in HaCaT cells. Cells were treated with CoQ_0_ (0-5 μM, 24 h). **A** Cell viability was examined by MTT assay. **B** Cells were subjected to immunoblotting to determine LC3-I/II, ATG4B, ATG5, and ATG7 protein levels. **C**, **D** Cells were first treated with or without 3-MA (1 mM, 1 h) followed by treatment with CoQ_0_ (0-5 μM, 24 h). Immunofluorescence staining was performed to determine LC3B levels. **E**, **F** Cells were treated with CoQ_0_ (0-5 μM, 24 h) and then subjected to immunoblotting to determine Beclin-1 and Bcl-2 protein levels. Data are presented as the Beclin-1/Bcl-2 ratio. The results are the mean ± SD (*n*=3). **p < 0.01; ***p < 0.001 compared with untreated cells. ^###^*p* < 0.001 compared with CoQ_0_-treated cells

We examined the cell viability of HaCaT cells treated with CoQ_0_. MTT assay showed that CoQ_0_ reduced HaCaT cell viability, with a maximum decrease in viability observed after 7.5 and 10 μM CoQ_0_ treatment (Fig. 10A). The results implied that CoQ_0_ concentration ≤ 5 μM was noncytotoxic for HaCaT cells. This dose was used for subsequent studies. The effect of CoQ_0_ (0-5 μM) on autophagy-associated proteins, such as LC3-I/II, ATG4B, ATG5, ATG7, Beclin-1, and Bcl-2, was tested in HaCaT cells. The immunoblotting data indicated that CoQ_0_ stimulation dose-dependently upregulated autophagic LC3-I/II protein expression (Fig. 10B). In addition, a decrease in the anti-autophagic protein ATG4B was observed, but increases in the pro-autophagic ATG5 and ATG7 proteins were observed in CoQ_0_-treated HaCaT cells (Fig. 10B). ATG5 is activated by ATG7 and combines with ATG12 and ATG16L1 to form a complex, and this complex is required for the conjugation of phosphatidylethanolamine to LC3-I to produce LC3-II [24]. Immunofluorescence staining confirmed that CoQ_0_ significantly increased LC3B expression, and 3-MA reversed CoQ_0_-mediated LC3B accumulation in HaCaT cells (Fig. 10C and D). Furthermore, CoQ_0_ treatment dose-dependently upregulated Beclin-1 expression but downregulated Bcl-2 expression (Fig. 10E and F). The Beclin-1/Bcl-2 ratio was significantly increased, leading to a shift in the cellular environment to promote autophagy in CoQ_0_-treated HaCaT cells (Fig. 10E and F). These data suggest that CoQ_0_ induces autophagy in HaCaT cells.

### CoQ_0_ induced autophagosome GFP-LC3 puncta and AVO formation in HaCaT cells

**Fig. 11:**
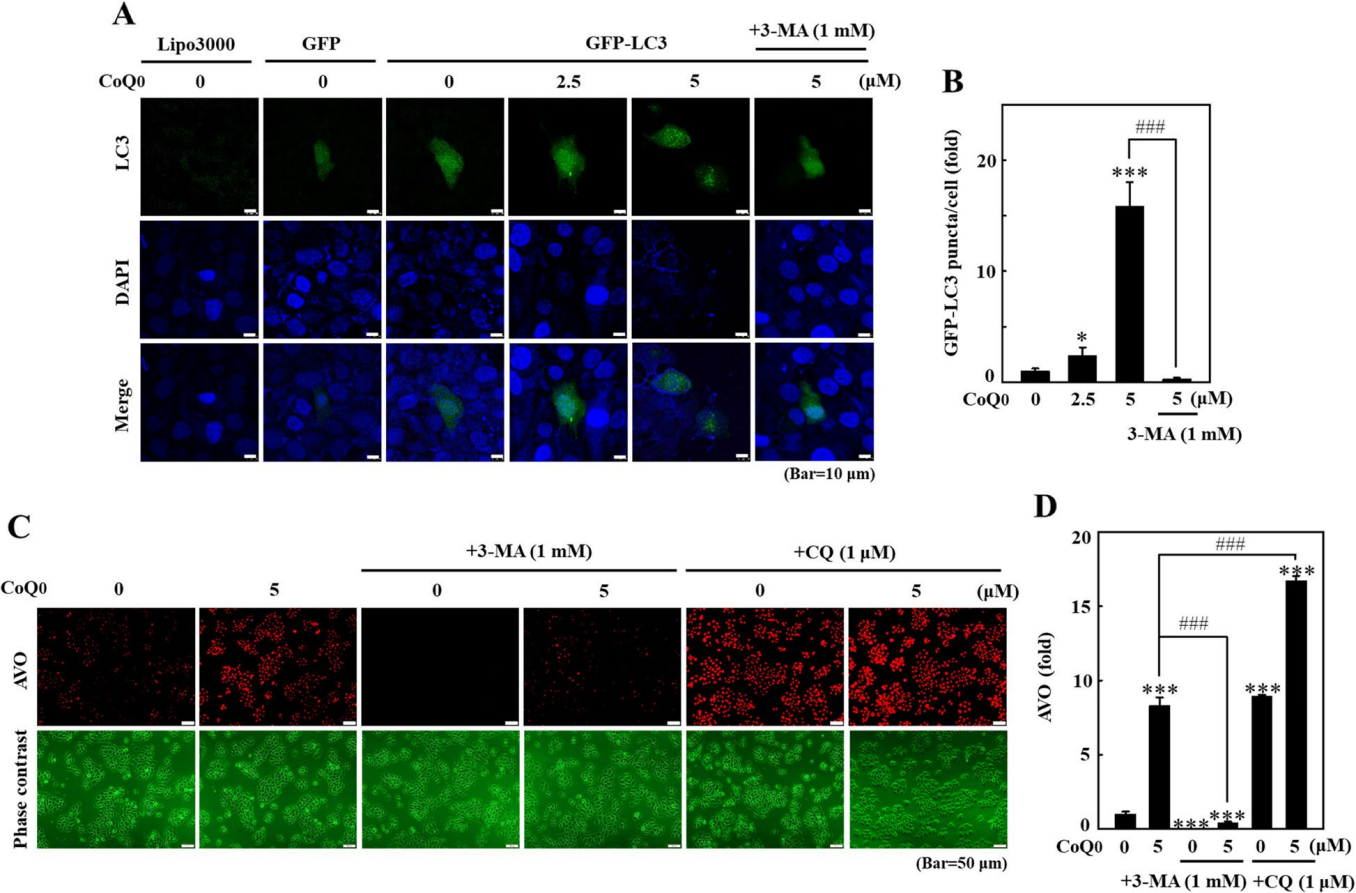
CoQ_0_ induced autophagy flux in HaCaT cells. Cells were first treated with or without 3-MA (1 mM, 1 h) and/or CQ (1 μM, 1 h) followed by CoQ_0_ (0-5 μM, 24 h). **A**, **B** Cells were transfected with the GFP-LC3 expression vector, and the formation of GFP-LC3 puncta induced by CoQ_0_ was observed under a confocal microscope. **C**, **D** Intracellular AVO formation in cells was observed under a fluorescence microscope. The results are the mean ± SD (*n*=3). *p < 0.05; ***p < 0.001 compared with untreated cells. ^###^*p* < 0.001 compared with CoQ_0_-treated cells

The GFP-LC3 plasmid was transiently transfected into HaCaT cells, and GFP-LC3 puncta were determined using confocal microscopy to further confirm CoQ_0_-induced autophagosome formation. CoQ_0_-treated (0-5 μM) HaCaT cells exhibited numerous green LC3 puncta in the cytoplasm (Fig. 11A and B). This effect was dose dependent, as the number of puncta increased with CoQ_0_ treatment (Fig. 11A and B). However, this effect was remarkably reversed in 3-MA-pretreated HaCaT cells, signifying that CoQ_0_ facilitated the formation of autophagosome GFP-LC3 puncta in HaCaT cells (Fig. 11A and B). Moreover, CoQ_0_-induced autophagy in HaCaT cells was further evaluated by detecting AVO formation. Fluorescence microscopy revealed that CoQ_0_ treatment resulted in significant AVO accumulation in HaCaT cells (Fig. 11C and D). However, preincubation with 3-MA (1 mM) diminished and CQ (1 μM) increased CoQ_0_-induced AVO formation (Fig. 11C and D). Thus, the CoQ_0_-mediated increase in AVO formation signifies autophagy induction in HaCaT cells.

### CoQ_0_ suppressed melanosome gp100 and melanin formation by inducing autophagy in melanin-feeding HaCaT cells

**Fig. 12:**
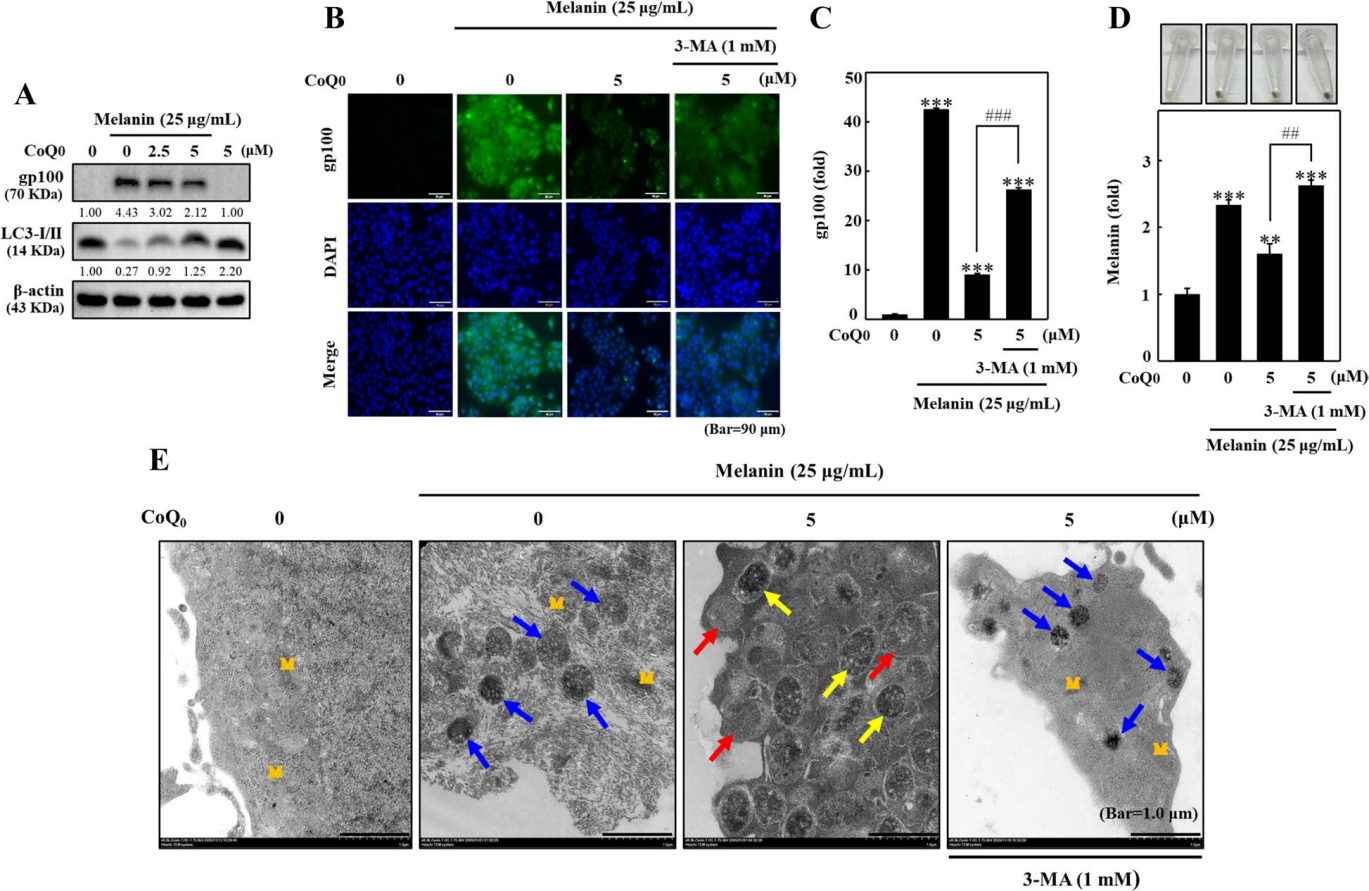
CoQ_0_ suppressed melanosome associated gp100 expression and triggered melanin degradation by inducing autophagy in melanin-feeding HaCaT cells. Melanin-treated HaCaT cells were pretreated with or without 3-MA (1 mM, 1 h), followed by treatment with CoQ_0_ (0-5 μM, 24 or 72 h). **A** The gp100 and LC3B expressions (24 h) were determined using immunoblotting. **B**, **C** The gp100 expression (24 h) as assessed by immunofluorescence staining. **D** The intracellular melanin levels (72 h) were estimated using the procedures described in the methodology section. The results are the mean ± SD (*n*=3). **p < 0.01; ***p < 0.001 compared with untreated cells. ^##^*p*< 0.01; ^###^*p* < 0.001 compared with CoQ_0_-treated cells. **E** TEM was used to analyze the CoQ_0_ promoted formation of melanosome-engulfing autophagosomes, and autolysosomes. Melanin-treated HaCaT cells were pretreated with or without 3-MA (1 mM, 1 h) followed by CoQ_0_ (0 or 5 μM, 24 h). M = mitochondria. The blue, yellow, and red arrows indicate melanin/melanosomes, autophagosomes containing melanin/melanosomes, and autolysosomes, respectively

Melanin is produced by melanocytes and kept in specialized organelles named melanosomes. Melanin is then transferred to nearby keratinocytes by these melanosomes [25]. Melanosomes, lysosome-related organelles, express the transmembrane melanoma antigen protein gp100. Notably, gp100 participates in the development of melanosomes [25]. HaCaT cells were preincubated with melanin (25 µg/mL) followed by CoQ_0_ treatment (0 or 5 μM for 24 h). The effect of CoQ_0_ on melanosome gp100 and LC3B expression was evaluated. Immunoblotting data showed that melanin-fed HaCaT cells exhibited upregulated gp100 expression and downregulated LC3B levels (Fig. 12A). Interestingly, CoQ_0_ treatment downregulated gp100 expression and upregulated LC3B levels (Fig. 12A). Immunofluorescence staining confirmed that CoQ_0_ significantly decreased gp100 levels in melanin-treated HaCaT cells (Fig. 12B and C). Pretreatment with 3-MA restored CoQ_0_-mediated gp100 suppression, suggesting the importance of autophagy in CoQ_0_-enhanced melanin degradation in melanin-feeding HaCaT cells (Fig. 12B and C). Consistent with the immunofluorescence staining and immunoblot data, CoQ_0_ induced melanin degradation, and pretreatment with 3-MA restored CoQ_0_-induced melanin degradation in melanin-feeding HaCaT cells (Fig. 12D).

### CoQ_0_ suppressed melanosome-engulfing autophagosome and autolysosome formation in melanin-feeding HaCaT cells

CoQ_0_-induced formation of melanosome-engulfing autophagosomes and autolysosomes in melanin-feeding HaCaT cells was analyzed using TEM to decipher the role of autophagy. As shown in Fig. 12E, melanosomes formed in melanin-feeding HaCaT cells. However, in the presence of CoQ_0_ (5 μM), autophagosomes engulfed melanosomes and autolysosome formation was observed in melanin-feeding HaCaT cells (Fig. 12E). Remarkably, this effect was reversed by 3-MA pretreatment (Fig. 12E). Therefore, CoQ_0_ induces autophagic flux in melanin-feeding HaCaT cells, leading to melanin degradation.

### CoQ_0_ provoked antimelanogenesis in zebrafish embryos

**Fig. 13:**
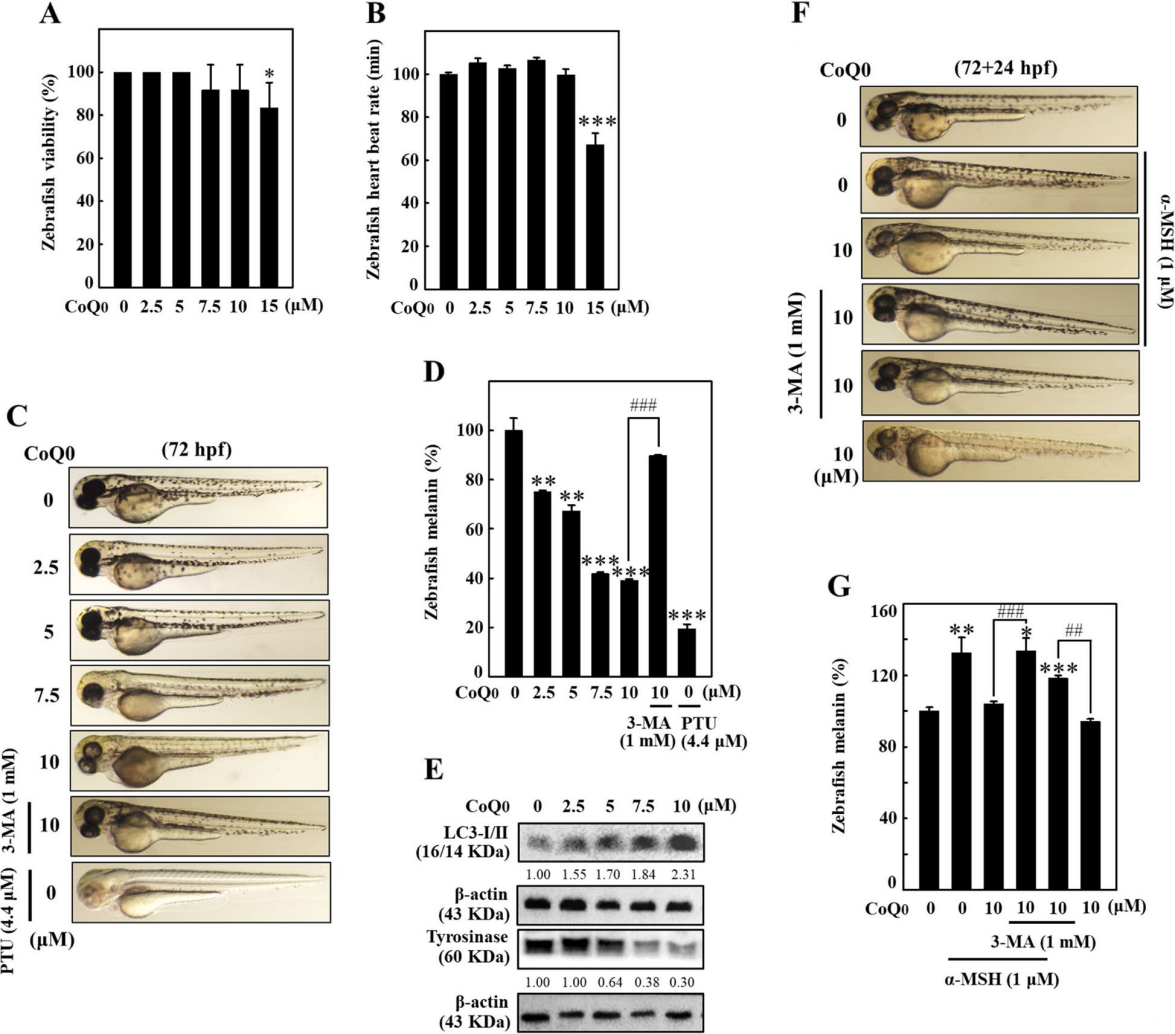
CoQ_0_ triggered antimelanogenesis and melanin degradation in zebrafish embryos. **A-E** Zebrafish embryos, 9 hpf, were treated with or without 3-MA (1 mM), followed by vehicle (0.1% DMSO) or CoQ_0_ (0-15 μM) up to 72 hpf. **A**, **B** The viability (%) and heart rate (beats/min) of zebrafish were measured using a stereomicroscope. **C**, **D** CoQ_0_ suppressed melanogenesis in zebrafish. PTU (4.4 μM) served as a positive control. **E** At the end of treatments, proteins were extracted, and an immunoblotting assay was conducted to measure LC3-II and tyrosinase expression. **F**, **G** CoQ_0_ triggered melanin degradation in zebrafish. Zebrafish at 72 hpf were treated with or without 3-MA (1 mM) followed by CoQ_0_ (0 or 10 μM) for 24 h (72+24 hpf) with or without α-MSH (1 μM) stimulation. The change in endogenous body pigmentation (melanin levels) in zebrafish (lateral views are shown) was measured as described in the methodology section. The results are the mean ± SD (*n*=3). *p < 0.05; **p < 0.01; ***p < 0.001 compared with control zebrafish embryos. ^##^*p* < 0.01; ^###^*p* < 0.001 compared with CoQ_0_-treated zebrafish

The in vivo antimelanogenesis effect of CoQ_0_ was further examined using zebrafish embryos as an experimental model. The effects of CoQ_0_ (0-15 μM at 9 hpf) on the viability and heart rate of zebrafish embryos at 72 hpf were first determined. Viability and heartbeat data measured through a stereomicroscope indicated that increasing concentrations of CoQ_0_ (up to ≤10 μM) did not exert significant effects on the viability and heart rate of zebrafish at up to 72 hpf, suggesting that testing **≦**10 μM CoQ_0_ in the context of in vivo experimentation is safe (Fig. 13A and B). Later, CoQ_0_ (0-10 μM at 72 hpf)-mediated endogenous body pigmentation and melanin formation were measured in zebrafish. Stereomicroscopic and melanin assay data showed that CoQ_0_ dose-dependently decreased endogenous body pigmentation and melanin formation in 72 hpf zebrafish (Fig. 13C and D). Interestingly, preexposure to 3-MA (1 mM 3-MA+10 μM CoQ_0_) significantly reversed CoQ_0_-mediated antimelanogenesis in zebrafish (Fig. 13C and D). Moreover, the proteins derived from zebrafish (72 hpf) were subjected to immunoblotting, and the results suggested that CoQ_0_ dose-dependently increased LC3B (favoring autophagy) and decreased tyrosinase (indicating antimelanogenesis) expression (Fig. 13E). PTU (4.4 μM) was used as a positive control. The results confirmed that autophagy plays a critical role in CoQ_0_-induced antimelanogenesis.

### CoQ_0_ triggered melanin degradation in zebrafish

To validate the in vitro depigmentation effects of CoQ_0_ in vivo, 72 hpf zebrafish were pretreated with or without 3-MA (1 mM) and/or α-MSH (1 μM), and then exposed to CoQ_0_ (0 or 10 μM) for 24 h (72+24 hpf). Compared to the control zebrafish, CoQ_0_ treatment markedly reduced α-MSH-stimulated endogenous body pigmentation and melanin formation in zebrafish (α-MSH+CoQ_0_) (Fig. 13F and G). However, this effect was reversed in zebrafish pretreated with 3-MA (3-MA+α-MSH+CoQ_0_) (Fig. 13F and G). The in vivo data suggested that CoQ_0_ provoked melanin degradation via autophagy in the zebrafish model.

## Discussion

**Fig. 14:**
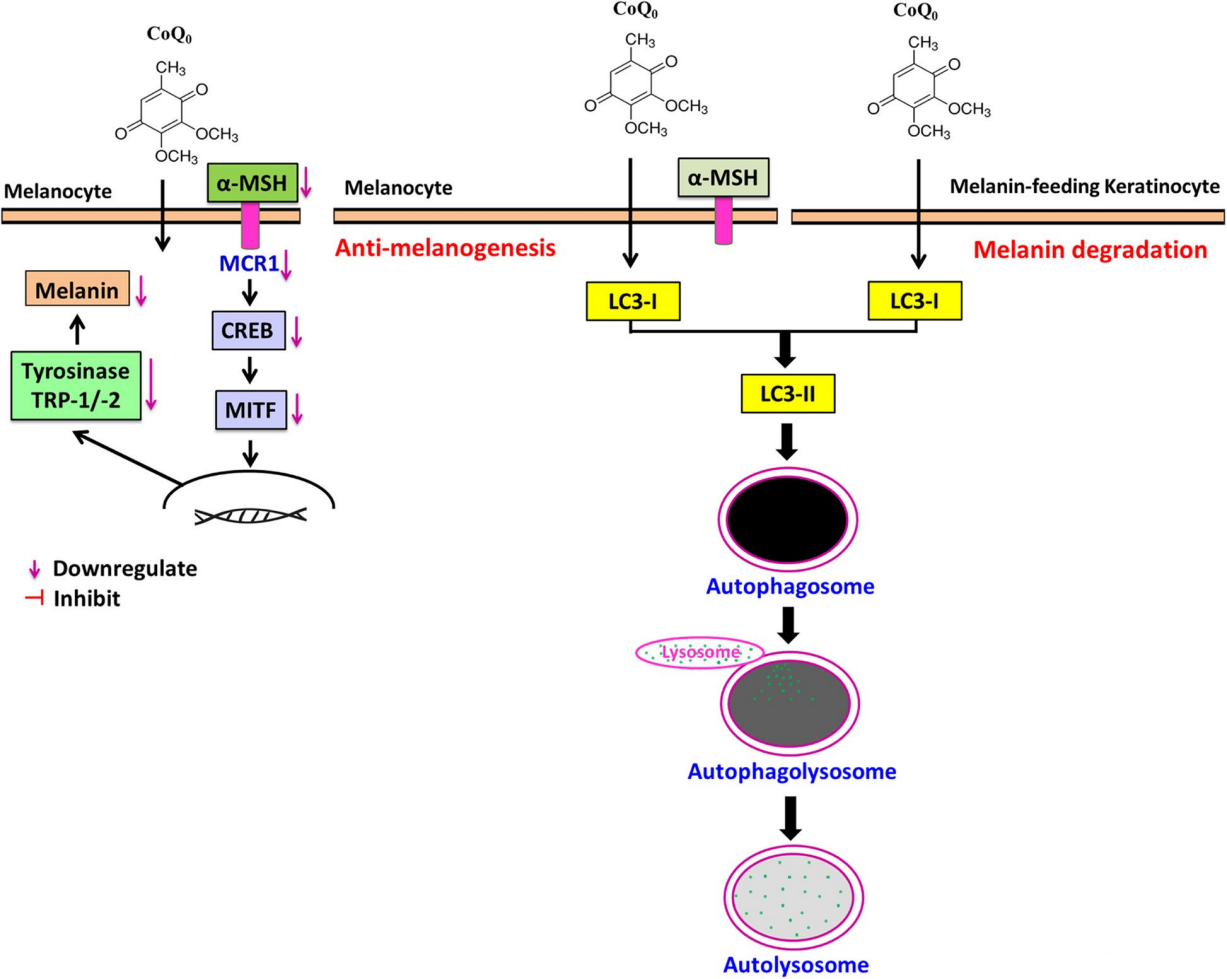
Depigmentation activity of CoQ_0_ through antimelanogenesis and melanin degradation by autophagy induction in melanoma (B16F10) and melanin-feeding keratinocyte (HaCaT) cells

Cosmetic compounds with depigmenting potential are in high demand for skin beautification and lightening. Therefore, the identification of new depigmenting compounds that possess antimelanogenic properties is urgently needed [26]. However, due to insufficient scientific evidence regarding the risks (carcinogenicity, allergies, and other side effects), many consumers have serious concerns about their usage. These compounds may be derived from natural sources for safety. CoQ_0_, a major quinone derivative from *Antrodia camphorata*, is a redox-active ubiquinone compound often found as a component of the mitochondrial respiratory chain [14]. In this study, we elucidated the autophagy mediated depigmentation activity of CoQ_0_ in in vitro (B16F10 and melanin-feeding keratinocyte HaCaT cells) and in vivo (zebrafish model) (Fig. 14).

The use of cosmetic products or ingredients in animal studies is forbidden. In Taiwan, Europe, and other countries, animal tests create ethical concerns. Zebrafish embryos are a common vertebrate model system in biochemical investigations because they exhibit many physiological and genetic similarities to mammals [27]. In depigmentation studies, the effects of cosmetic ingredients on the skin/surface of a zebrafish are easy to observe. In the present study, CoQ_0_ decreased endogenous body pigmentation and melanin formation in zebrafish by inducing autophagy. Moreover, CoQ_0_ (up to ≤10 μM) did not affect the viability and heartbeat of zebrafish, indicating that there is no toxicity to CoQ_0_-treated zebrafish. Consistent with the in vitro results, the in vivo data derived from 3-MA application confirmed that autophagy plays a pivotal role in CoQ_0_-triggered antimelanogenesis and melanin degradation. As a result, the screening of zebrafish embryos for CoQ_0_ incited antimelanogenesis and melanin degradation replaced animal trials for the use of CoQ_0_ in cosmetic products or ingredients.

In melanocytes, the mechanism of α-MSH-stimulated melanogenesis is well defined. A number of transcription factors, including CREB and MITF, are involved in melanogenesis [2]. Tyrosinase and TRP-1/-2 are crucial melanogenic enzymes. Enzyme transcription is closely controlled by MITF, and MITF is thus viewed as a master regulator of melanogenesis [3]. Our results showed that CoQ_0_ triggered antimelanogenic effect was regulated by the MC1R-cAMP-CREB-MITF pathways. In unstimulated or α-MSH-stimulated B16F10 cells, CoQ_0_ repressed MC1R/CREB/MITF, tyrosinase, and TRP-1/-2 expression and melanin production, suggesting that CoQ_0_ exerted strong antimelanogenesis effects on melanocytes. Interestingly, CoQ_0_ suppressed MITF nuclear translocation via the ERK, JNK, and PI3K/AKT pathways, suggesting the complexity of the melanogenesis process. ERK activation enhances the antimelanogenic effect in α-MSH-stimulated melanocytes [2]. MITF instability and degradation are caused by activated ERK phosphorylating MITF at Ser73. JNK inhibits MITF production through CREB pathways [28]. As has been previously discovered, JNK activation inhibits the CREB-MITF signaling pathway which could be enhanced pharmacologically to suppress melanogenesis. PI3K/AKT signaling is an important antimelanogenic pathway and is frequently activated in melanoma cells [29]. PI3K/AKT activation and subsequent GSK3β inhibition provoke antimelanogenesis through dephosphorylation of MITF at Ser298 which inhibits binding to tyrosinase and TRP-1/-2 promoters and decrease melanin formation [30]. Our findings were consistent with previous studies implicating the important roles of ERK, JNK, and PI3K/AKT activation in the inhibition of MITF nuclear translocation, and therefore anti-melanogenesis.

The development of autophagy-inducing agents offers latent clinical benefits for the treatment of diseases and for the inhibition of abnormal skin pigmentation by melanocytes and keratinocytes [31]. Autophagy is a conserved self-catabolic process that helps protect the cellular microenvironment from different stresses; thus, basal autophagy is necessary for cellular homeostasis. The contribution of autophagy to melanocyte and keratinocyte biology has already been documented [7]. Moreover, earlier studies suggested that autophagy regulators and melanosome biogenesis-associated regulators have overlapping molecular mechanisms [31]. In the current study, we verified the efficacy of CoQ_0_ in inducing autophagy mediated antimelanogenesis in B16F10 and HaCaT cells. CoQ_0_-induced autophagy in B16F10 and HaCaT cells was shown by enhanced LC3-II accumulation, ATG4B downregulation, autophagosome GFP-LC3 puncta and AVO formation, and Beclin-1/Bcl-2 dysregulation. However, pretreatment with 3-MA reversed these activities, suggesting that antimelanogenic effect of CoQ_0_ is mediated via autophagy in B16F10 and HaCaT cells.

Melanocytes and keratinocytes interact during melanogenesis and melanin formation [31]. Our results revealed that CoQ_0_ treatment did not markedly alter melanocyte and keratinocyte viability, indicating that CoQ_0_ is safe for those skin cells. Autophagy-mediated antimelanogenesis was reported to affect physiological skin color [31]. In α‐MSH-stimulated B16F10 cells, CoQ_0_ triggered antimelanogenesis by inhibiting p-MITF, tyrosinase expression/activity, and melanin formation via autophagy. TEM provided evidence of melanosome engulfment by autophagosomes, and autolysosomes formation, following CoQ_0_ treatment in α‐MSH-stimulated B16F10 cells. Notably, these CoQ_0_-mediated antimelanogenesis effects were reversed in 3-MA-pretreated cells. LC3 silencing also significantly suppressed the expression of early- and late-stage melanosome marker proteins and melanin levels in LC3-silenced cells. These results cumulatively indicated that autophagy induction by CoQ_0_ negatively affects the melanogenesis and hyperpigmentation in skin cells.

Melanosomes, which are lysosome-related organelles, are where melanin is kept after being produced by melanocytes and transferred to nearby keratinocytes. Physiological skin color is influenced by the balance between the quantity of melanosomes and the level of phagocytic activity of keratinocytes that feed on melanin [16]. Melanosomes express the transmembrane antigen protein gp100, and gp100 is involved in melanosome maturation [25]. Initial data showed that in melanin-feeding HaCaT cells, CoQ_0_ inhibited melanosome associated gp100 expression. Notably, CoQ_0_ inhibited melanin formation in melanin-feeding HaCaT cells. Further analyses using TEM revealed that CoQ_0_ enabled autophagosomes to engulf melanosomes and autolysosome formation in melanin-feeding HaCaT cells. However, CoQ_0_ mediated melanin degradation, which was restored in 3-MA pretreated cells. The results confirmed that CoQ_0_ promoted autophagic flux, leading to melanin degradation in melanin-feeding HaCaT cells. From our results, the primary target of CoQ_0_ appears to be cell surface expression of MC1R which exhibited significantly decreased levels by CoQ_0_ treatment (0-5 μM) in B16F10 cells. There were concomitant reduced expressions of MC1R downstream signaling mediators including p-CREB, CREB, MITF, and tyrosinase. However, previous researches have suggested that MC1R expression and melanogenesis is also impacted by environmental signals (UV-R, cellular stresses) and various growth factor signaling (ET-1, FGF etc.,) [32, 33]. Therefore, further studies are required to ascertain the precise molecular mechanisms of CoQ_0_ in anti-melanogenesis/melanin degradation.

## Conclusions

This investigation is the first to demonstrate that CoQ_0_, a major quinone derivative from *Antrodia camphorata*, exerts autophagy mediated depigmenting effects on keratinocytes and melanocytes. In B16F10 and melanin-feeding HaCaT cells, CoQ_0_ exerted antimelanogenesis and melanin degradation effects by inducing autophagy. The in vivo results confirmed that CoQ_0_ inhibited endogenous body pigmentation in zebrafish embryos by instigating autophagy. Altogether, our findings suggested that CoQ_0_ might be used as a depigmenting ingredient in the skin care formulations.

### Supplementary Information

**Supplementary Material 1.**

## Data Availability

The datasets used and/or analyzed during this study are included within the manuscript and would be available from the corresponding author on reasonable requests.

## Abbreviations

α-MSH: α-melanocyte stimulating hormone
MC1R: Melanocortin 1 receptor
MITF: Microphthalmia-associated transcription factor
CREB: cAMP response element-binding protein
TRP-1/-2: Tyrosinase-related protein-1/-2
LC3: Microtubule-associated protein light chain 3
ATG4B: Autophagy-related 4B cysteine peptidase
ATG5: Autophagy-related protein 5
ATG7: Autophagy-related protein 7
AVO: Acidic vesicular organelle
3-MA: 3-methyladenine
CQ: Chloroquine
TEM: Transmission Electron Microscopy
gp100: Glycoprotein 100
PTU: Phenylthiourea
