# Supplementary material for: The in vitro and in vivo depigmentation activity of coenzyme Q0, a major quinone derivative from Antrodia camphorata, through autophagy induction in human melanocytes and keratinocytes
Source: Cell Commun Signal. 2024 Feb 26;22:151. doi: 10.1186/s12964-024-01537-6 (PMC10895752; doi:10.1186/s12964-024-01537-6)
Supplement: Supplementary file 1 — Supplementary Material 1. [file 12964_2024_1537_MOESM1_ESM.docx]

**Highlights**

• CoQ_0_, a quinone derivative of *Antrodia camphorata*, inhibits melanogenesis in melanoma B16F10 cells

• CoQ_0_ induced autophagy in melanoma B16F10 and keratinocyte HaCaT cells

• CoQ_0_ triggered antimelanogenesis via autophagy in α‐MSH-stimulated B16F10 cells

• CoQ_0_ enhanced melanin degradation via autophagy in melanin-feeding HaCaT cells

• CoQ_0_ suppressed endogenous body pigmentation via autophagy in the *in vivo*zebrafish
